# Longitudinal Changes in Auditory and Cognitive Function in Middle-Aged and Older Adults

**DOI:** 10.1044/2020_JSLHR-20-00274

**Published:** 2021-01-05

**Authors:** Larry E. Humes

**Affiliations:** a Department of Speech, Language and Hearing Sciences, Indiana University Bloomington

## Abstract

**Purpose:**

This article aimed to document longitudinal changes in auditory function, including measures of temporal processing, and to examine the associations between observed changes in auditory and cognitive function in middle-aged and older adults.

**Method:**

This was a prospective longitudinal study of 98 adults (66 women) with baseline ages ranging from 40 to 85 years. The mean interval between T1 baseline and T2 follow-up measurements was 8.8 years with a range of 7–11 years. Measures of hearing threshold, gap detection, and auditory temporal-order identification were completed at T1 and T2. Cognitive measures completed at T1 and T2 were the 13 scales of the Wechsler Adult Intelligence Scale–Third Edition. Three approaches were taken to analyze these data: (a) examination of changes over time in group performance, (b) correlations and slopes between auditory and cognitive measures to examine concomitant *rates* of decline over the 9-year T1-to-T2 period, and (c) regression analyses examining associations between auditory performance at T1 and cognitive performance 9 years later at T2.

**Results:**

For the group data, there were significant declines in hearing loss, gap-detection thresholds at one frequency, and process-type measures of cognitive function from T1 to T2 matching the trends in the baseline cross-sectional data. Regression analyses of the longitudinal data revealed the strongest connection between auditory temporal-order processing and cognitive processing typically explaining 10%–15% of the variance.

**Conclusions:**

A significant amount of variance in rates of cognitive decline, T1 to T2, and subsequent cognitive performance (T2) was explained by measures of auditory function. Although hearing loss occasionally emerged as a significant factor, auditory temporal-order identification emerged much more frequently as the auditory measure most strongly associated with cognitive function.

For many years, there has been interest in the concomitant changes in sensory and cognitive function that accompany aging (e.g., Humes & Young, 2016; Schneider & Pichora-Fuller, 2000; Wayne & Johnsrude, 2015). As noted by Humes, Busey, et al. (2013), most prior studies of this association involved one sense, most commonly either vision or hearing, only occasionally including both senses. Typically, simple measures of sensory acuity, such as the audiogram for hearing, were the only measures included for the sense under study, although speech-in-noise measures have been used more recently in studies of age-related auditory and cognitive changes (Pronk et al., 2019; Ronnberg et al., 2014). Humes, Busey, et al. (2013) included multiple psychophysical measures in three senses: hearing, vision, and touch. The psychophysical measures included sensitivity thresholds and a variety of temporal-processing measures. In that cross-sectional study of 245 young, middle-aged, and older adults, moderate associations among aging, sensory processing, and cognitive function were observed.

The data in Humes, Busey, et al. (2013) were cross-sectional in nature. Longitudinal designs offer the possibility of obtaining stronger evidence of cause-and-effect associations among measures than is generally possible with cross-sectional designs (Evans, 1978; Schaie, 1983, 2005). Although there have been several longitudinal studies of hearing thresholds measured clinically via audiometry, there appears to be only one other longitudinal study of auditory temporal processing in adults (Babkoff & Fostick, 2017). Babkoff and Fostick (2017) measured dichotic temporal-order judgment for 15-ms, 1000-Hz pure tones and observed a longitudinal decline over the age range of 22–82 years (*N* = 58). No such decline was observed in the same participants for gap-detection threshold for a 1000-Hz pure tone.

From the prior cross-sectional study (Humes, Busey, et al., 2013), the links between sensory function and cognitive processing were greatest for the measures of temporal processing compared to simple measures of hearing threshold (Danielsson et al., 2019; Humes, Busey, et al., 2013). The focus was placed on temporal processing in Humes, Busey, et al. (2013) because of mounting evidence supporting age-related changes in such processing that were independent of peripheral hearing declines but important contributors to cognitive function and to speech communication in older adults (Humes & Dubno, 2010; Humes et al., 2012; Humes, Kidd, & Lentz, 2013).

To gather longitudinal data for the auditory and cognitive measures, 203 older and middle-aged adults from Humes, Busey, et al. (2013) were recruited for participation in an 8- to 9-year follow-up study. One of the potential hazards to the interpretation of longitudinal measures is the practice or learning that can take place with frequent repetition of the tests over time. Salthouse (2011) examined test–retest intervals of 1–8 years for cognitive function in adults and found that an 8-year interval was sufficient to minimize or eliminate such concerns about practice effects. This consideration led to the use of an 8- to 9-year interval between baseline and follow-up measurements here.

This report provides the results for 98 adults, 48.3% of the original cohort, who returned for the 8- to 9-year longitudinal follow-up study. The specific auditory measures included (a) clinical measures of hearing threshold from 250 to 8000 Hz bilaterally; (b) psychophysical measures of hearing threshold at 500, 1400, and 4000 Hz; (c) psychophysical measures of gap-detection threshold for 1000-Hz bands of noise centered at 1000 and 3500 Hz; and (d) four measures of temporal-order identification of brief vowel sequences presented either monaurally or dichotically. The reason both clinical and psychophysical measures of hearing threshold were included at baseline and follow-up owes to the long history of debate about sensitivity versus criterial differences between the hearing thresholds of young versus older adults when measured clinically (Gatehouse & Davis, 1992; Marshall, 1991; Marshall & Jesteadt, 1986; Potash & Jones, 1977; Rees & Botwinick, 1980). In addition to these auditory measures, the full Wechsler Adult Intelligence Scale–Third Edition (WAIS-III; Wechsler, 1997) was obtained as the cognitive assessment, as in the baseline study.

The results for the baseline (T1) and 9-year follow-up (T2) measures will be examined in several ways below, each analysis addressing slightly different questions. First, are there significant changes in auditory and cognitive function in this cohort of 98 adults from T1 to T2? This is primarily addressed by comparison of group mean performance from T1 to T2 with individual differences in the changes over time examined via correlations between T1 and T2 performance. As will be seen below, moderate and significant correlations in auditory and cognitive performance over time were observed. This then permitted the calculation of linear slopes for the *rates* of change in auditory and cognitive function from baseline T1 to 9-year follow-up T2 measures. Here, the question addressed is whether the *rate* of decline in auditory function over this 9-year period is associated with the *rate* of decline in cognitive function in the same individuals over this same 9-year period. Such associations would support common underlying mechanisms for both auditory and cognitive declines with advancing age (e.g., Lindenberger & Baltes, 1994). The final question addressed below is whether auditory function at baseline (T1) is predictive of subsequent cognitive function 9 years later (T2). If so, then this argues in favor of mechanisms which model auditory decline as a precursor to cognitive decline, such as various sensory-deprivation or information-degradation models (Schneider & Pichora-Fuller, 2000; Wayne & Johnsrude, 2015). This question will be addressed primarily through a series of multiple-regression analyses examining associations between the measures of T1 auditory function and T2 cognitive function.

## Method

### Participants

The pool of potential participants who were in the older or middle-aged group at baseline was composed of the 135 older adults and 60 middle-aged adults included in Humes, Busey, et al. (2013) and an additional eight individuals (six older, two middle-aged) who completed the baseline measures after publication of the results in 2013. This resulted in a total pool of 203 prospects. The 50 young adults included in Humes, Busey, et al. (2013) were not recruited as most were college undergraduates at baseline and no longer lived nearby. Of the 203 prospects, 99 declined to participate. For these 99 nonparticipants, the four primary reasons for not returning for follow-up were that the participant died before recruitment for the follow-up study (26.3%); staff were unable to contact the prospect by phone, mail, or e-mail (24.2%); ongoing physical health or mobility restrictions (15.2%); or the participant had moved away (12.1%). It should be noted that cognitive concerns could also be indicated as a reason for declining to participate, but this was indicated in only four of 99 (4%) cases. A total of 104 of the baseline T1 participants returned for the T2 follow-up, but six (five older, one middle-aged) withdrew from the follow-up study for health reasons prior to completion and their data are not included in the T2 follow-up results.

A total of 98 adults (66 women, 32 men) with a mean age of 62.6 years (range of 40–85 years) at baseline participated in this longitudinal follow-up study. These participants ranged in age at follow-up from 47 to 94 years, with a mean age of 71.5 years, at follow-up. Most (76.5%) were retested 9 years following baseline, an additional 14.3% within 1 year of the 9-year retest interval, with the remainder retested at either a 7-year (8.2%) or 11-year (1.0%) interval. The mean interval between T1 baseline and T2 follow-up measurements was 8.8 years. Given these variations in test–retest or T1–T2 interval, this will often be treated as a covariate in several of the analyses to follow.

**Figure 1.**
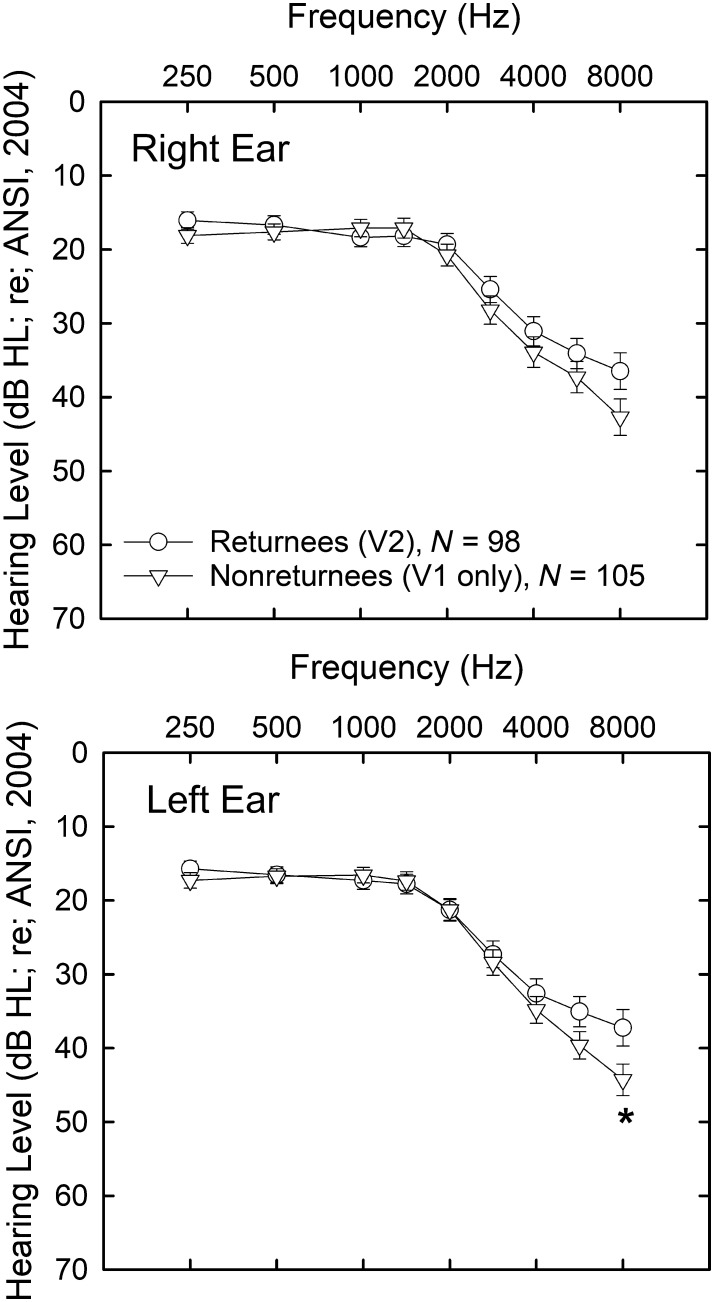
Means and standard errors of air-conduction audiometric thresholds for returnees (circles) and nonreturnees (triangles) from original baseline (T1) study. Threshold data for the right ear are provided in the top panel and left ear in the bottom panel. The asterisk for the hearing threshold at 8000 Hz in the left ear marks the only significant (*p* < .05) difference in thresholds between the two groups.

On most baseline (T1) measures included in the longitudinal follow-up study, there were no significant differences (*p* > .05; independent-samples *t* tests, uncorrected for multiple comparisons) between the 98 returnees who completed the follow-up measures and the 105 who either did not return (*N* = 99) or did not complete the follow-up study (*N* = 6). For convenience, the latter group of 105 have been designated here as “nonreturnees.” Figure 1 shows the baseline audiograms for right and left ears for these two groups, and the only significant difference between the two groups occurred at 8000 Hz in the left ear. Similarly, the two groups did not differ significantly (*p* > .05) in age at baseline evaluation, with the returnees having a mean age at baseline of 62.6 years and the nonreturnees at 65.3 years. The two groups also did not differ significantly regarding their baseline scores on the Mini-Mental State Exam (MMSE; Folstein et al., 1975). The two groups *did* differ significantly (chi-square test, *p* < .05) regarding the proportion of men and women: 54 of 105 nonreturnees (51%) and 66 of 98 (67%) of the returnees were women. Regarding baseline differences among the auditory and cognitive measures described in more detail below, non-returnees had significantly worse baselines for auditory gap-detection threshold at 1000 Hz, raw block-design WAIS-III subscale scores, and raw matrix-reasoning WAIS-III subscale scores. All told, these differences amount to significant differences (*p* < .05) in baseline performance between returnees and nonreturnees on only one of 18 pure-tone audiometric thresholds, one of nine auditory psychophysical measures, and two of 13 WAIS-III subscale scores. It is noteworthy, though, when significant group differences were observed in baseline scores, it was always with the 98 returnees outperforming the 105 nonreturnees. Nonetheless, the two groups were much more similar than dissimilar and we conclude that the 98 returnees are a representative sample of the original baseline cohort of 203 adults.

Informed consent was obtained from all 98 participants, and they were paid $12/hr for their participation. This study was approved by the Indiana University Bloomington Institutional Review Board.

### Materials and Procedures

A general objective of the baseline study was to obtain a comprehensive set of threshold sensitivity and temporal-processing measures in hearing, vision, and touch, using identical psychophysical procedures and similar stimuli for each sense. Given that the original study involved about 40 sessions, each 90-min in length, we decided to abbreviate the battery of tests included in the longitudinal follow-up study. First, we opted to drop the tactile measures in the follow-up, focusing on the senses of hearing and vision instead. In addition, the auditory measures of temporal masking used in the original study proved to be largely redundant with the measures of temporal-order identification. These measures were also eliminated for the T2 follow-up study. Here, we report on only the auditory measures of sensory function.

For each of the remaining psychophysical measures, as in the baseline study, a “threshold estimate” of performance was preceded by 20–40 familiarization trials, which included trial-to-trial feedback, and was obtained on the basis of three separate and stable blocks of trials that, when pooled, totaled 200–250 trials. The details of the stimuli and the psychophysical procedures for the auditory stimuli and procedures used here can be found in a series of prior studies (Fogerty et al., 2010; Humes et al., 2009, 2010). During the initial session of the follow-up study, audiological examinations were completed, along with the MMSE. The subjects next completed the full WAIS-III yielding the 13 standard scale scores. Raw WAIS-III scores, rather than age-corrected scores, are used throughout this report.

Next, the measures of auditory threshold sensitivity and gap detection were completed. For auditory threshold measurement, measures were obtained first at 500 Hz, then at 1400 Hz, and finally at 4000 Hz. Similarly, measurement of gap-detection threshold began at the 1000-Hz center frequency and then proceeded to the 3500-Hz center frequency. This use of a fixed order reinforced the need for familiarization trials prior to each measure and for stable threshold estimates based on 200–250 trials. Next, temporal-order identification measures were completed. Four temporal-order identification tasks were completed. Three of the four tasks required the identification of two-item sequences (out of the four possible stimuli), and one required the identification of a four-item sequence. The three 2-item sequences differed regarding how the stimuli were presented to the subject with stimuli in the sequence presented either to the same ear (monaural) or to different ears (dichotic). This manipulation was designed to explore lower level (peripheral) versus higher level (central) auditory temporal-processing mechanisms. For example, for the auditory two-item dichotic task, the two sensory inputs cannot interact until the first auditory center in the brainstem processes inputs from both ears (the superior olivary complex). On the other hand, the same-ear monaural version of this task makes it possible for interaction of the two stimuli in the sequence at a much lower level, as low as the cochlea. For the two dichotic, two-item tasks, the difference between them was in the response required of the subject. In one case, the subject was required to identify the stimulus sequence, just as in the monaural version of this task, whereas in the other case, the task was simply to identify which ear (right or left) was stimulated first. The latter temporal-order identification task was included because this is most often considered “temporal-order judgment” in the long history of interest in this measure (e.g., Fraisse, 1984; James, 1890). In addition, there have been some studies of the effects of aging on this form of temporal-order judgment (e.g., Babkoff & Fostick, 2017; Ronen et al., 2018). Finally, the monaural four-item sequence was included to increase the cognitive demands for this temporal-order identification task, thereby increasing the likelihood for uncovering a common underlying cognitive factor. For all these auditory temporal-order measures, the threshold estimate obtained was the stimulus onset asynchrony (SOA) that was approximately midway between chance and 100% correct performance on the psychometric function relating performance to SOA. Further details regarding the stimuli and procedures can be found elsewhere (Fogerty et al., 2010; Humes et al., 2010).

### Auditory Procedures and Equipment

All auditory psychophysical testing was completed in a sound-attenuating booth meeting the American National Standards Institute S3.1 standard for “ears covered” threshold measurements (American National Standards Institute, 2003). Two adjacent subject stations were housed within the booth. Each participant was seated comfortably in front of a touchscreen display (Elo Model 1915L). The right ear was the test ear for all monaural measurements in this study. Stimuli were generated off-line and presented to each listener using custom MATLAB software. Stimuli were presented from the Tucker-Davis Technologies (TDT) digital array processor with 16-bit resolution at a sampling frequency of 48828 Hz. The output of the digital-to-analog converter was routed to a TDT programmable attenuator (PA-5), TDT headphone buffer (HB-7), and then to an Etymotic Research 3A insert earphone. Each insert earphone was calibrated acoustically in an HA-1 2-cm^3^ coupler (Frank & Richards, 1991). Output levels were checked electrically just prior to the insert earphones at the beginning of each data-collection session and were verified acoustically using a Larson Davis Model 2800 sound-level meter with linear weighting in the coupler monthly throughout the study. Prior to actual data collection in each experiment, all listeners received 10–30 practice trials to become familiar with the task. These trials could be repeated a second time to ensure comprehension of the tasks, if desired by the listener, but this was seldom requested. All responses were made on the touchscreen and were self-paced. Correct/incorrect feedback was presented after each response during experimental testing. Further methodological details, specific to each measure, follow.

Auditory thresholds were measured for three pure-tone frequencies, 500, 1414, and 4000 Hz. Stimuli were 500 ms in duration from onset to offset and had 25-ms linear rise-fall times. The maximum output for the pure-tone stimuli was 98, 100, and 101 dB SPL at 500, 1414, and 4000 Hz, respectively. Further attenuation was provided via the programmable attenuator under software control during the measurement of auditory thresholds. Two auditory gap-detection measurements were made, each with a different 1000-Hz wide band of noise. These noise bands served as the stimuli with one band centered arithmetically at 1000 Hz (500–1500 Hz) and the other centered at 3500 Hz (3000–4000 Hz). Each noise band had a duration from onset to offset of 400 ms with 10-ms linear rise–fall times. A catalogue of 16 different noise bands was generated for each frequency region. Hanna and Robinson (1985) demonstrated that, if fewer than 10 samples of reproducible noise are used, listeners can make use of cues specific to a particular waveform and results may not generalize to true random noise. When a temporal gap was present in a noise band, it was centered at 300-ms post stimulus onset. This temporal location of the gap is more sensitive to age effects than a location centered in the noise stimulus (Harris et al., 2010). Gap durations varied from 2 to 40 ms in steps of 2 ms and were generated by zeroing the waveform at that temporal location, which necessitated the use of a background noise that covered a broad spectrum. This ensured that the cue available to the listener for gap detection was temporal and not spectral in nature. The spectrum level of the background noise was adjusted to be 12–15 dB below that of the stimulus noise bands. The background noise began slightly before the first interval and ended slightly after the last interval for a total duration of 2.4 s. An overall presentation level of 91 dB SPL was used for each noise band and for all listeners in this study. A relatively high presentation level was used given the likelihood of significant threshold elevations in many of the older adults, especially at the higher frequencies. Additional details of stimulus construction and calibration can be found in Humes et al. (2009).

Threshold measurements were completed prior to gap-detection measurements for all listeners. For measures of threshold sensitivity, an adaptive two-interval, two-alternative forced-choice paradigm was employed. Listeners simply selected the interval (marked by a rectangular box on a visual display) that contained the signal with an a priori probability of 0.5 that the signal would be in either Interval 1 or Interval 2. Signal amplitude was varied adaptively from trial to trial to bracket the 70.7% and 79.3% correct points on the psychometric function using two interleaved tracks (Levitt, 1971). Three estimates each of 70.7% and 79.3% correct performance were obtained for a given signal frequency. These six performance estimates were averaged to provide a single threshold estimate corresponding to approximately 75% correct on the psychometric function. For measures of gap-detection thresholds, gap duration was varied using the same interleaved adaptive tracking procedures as those described for the threshold measurements, including performance levels tracked (70.7% and 79.3%). In addition, for these measurements, a three-interval, two-alternative forced-choice paradigm was used as described more fully in Humes et al. (2009). The stimulus waveforms in a given trial were identical except that a temporal gap had been inserted into the stimulus presented during Comparison Intervals 1 or 2. The specific noise-band waveform used on a given trial, however, was randomly selected among the 16 available in a stimulus catalogue. The listener's task on each trial was to select the comparison interval that contained the gap or that differed from the standard (which never contained a gap).

For the four auditory temporal-order identification tasks, four confusable vowel stimuli /I, e, a, u/ were recorded by a male talker in a sound-attenuating booth using an Audio-Technica AT2035 microphone. Vowels were produced in a /p/-vowel-/t/context. Productions of four vowels that had the shortest duration, F2 < 1800 Hz, and good identification during piloting were selected for stimuli. Stimuli were digitally edited to remove voiceless sounds, leaving only the voiced pitch pulses, and modified in MATLAB using STRAIGHT (Kawahara et al., 1999) to be 70 ms long with a fundamental frequency of 100 Hz. Stimuli were low-pass filtered at 1800 Hz and normalized to the same root-mean-square level. Low-pass filtering was used to minimize the influence of possible high-frequency hearing loss of the older adults on their vowel-identification performance. The system was calibrated using a calibration vowel of the same root-mean-square amplitude as the test stimuli, but with a duration of 3 s. A single stimulus presentation measured 83 (±2) dB SPL and a presentation of two overlapping stimuli measured 86 (±2) dB SPL.

All listeners completed the four temporal-order tasks in the following order: monaural two-item identification (Mono2), monaural four-item identification (Mono4), dichotic two-item vowel identification (DichID), and dichotic two-item ear or location identification (DichLOC). For all four tasks, the same vowel was never repeated twice in a row. The Mono4 task had the additional stipulation that each sequence must contain at least three of the four vowel stimuli. For the three vowel-identification tasks, listeners were required to identify, using a closed-set button response, the correct vowel sequence exactly (i.e., each vowel in the order presented) for the response to be judged correct. The ear-identification task, DichLOC, only required the listener to identify which ear (“Right” or “Left”) was stimulated first. The dependent variable measured was the SOA between the presented vowels. The minimum SOA values were required to begin at or above 2 ms to ensure a sequential presentation for the stimuli. Given the 70-ms stimulus duration, any SOA values less than 70 ms involved varying degrees of temporal overlap among successive stimuli. For the four-item sequences, the SOA defined the onset asynchrony between successive stimulus pairs in the sequence. For example, an SOA of 10 ms indicates that the onset of the second vowel followed the onset of the first vowel by 10 ms, the onset of the third vowel followed the second vowel by 10 ms, and the onset of the fourth vowel followed the onset of the third vowel by 10 ms. Again, SOAs less than 70 ms involved varying degrees of temporal overlap among the stimuli in a sequence. All temporal-order tasks used the method of constant stimuli to measure the psychometric function relating percent correct identification performance to SOA. Threshold was defined as 50% correct (75% correct for DichLOC given two possible responses). Experimental testing was conducted in two stages because of large variability between listeners. The first stage consisted of a preliminary wide-range estimate of SOA threshold (i.e., using a large step size, 25 ms), while the second stage consisted of narrow-range testing centered at an individual's estimated wide-range threshold (i.e., using a smaller step size, 10 or 15 ms) to provide the actual SOA threshold estimates reported in the results. In the end, each threshold estimate for each temporal-order task was based on three valid narrow-range estimates that were averaged together for analysis, resulting in a total of 216 (Mono2), 288 (Mono4), or 432 (DichID, DichEar) trials per SOA threshold estimate.

### Data Analyses

Prior to data analyses, the results were examined for outliers for the nine psychophysical and the 13 WAIS-III cognitive measures. SPSS (Version 26) was used to identify major outliers. Major outliers were defined as falling more than 3 times the interquartile range above the third quartile or below the first quartile for that measure. For example, assume a first quartile for some measure of 75 ms and a third quartile on that same measure of 100 ms, then the interquartile range would be 25 ms and values less than 0 ms or greater than 175 ms would be considered to be major outliers. Three or fewer, of 98, data points were identified and disregarded as major outliers for 20 of the 22 measures with 0 major outliers identified for 14 of the 22. Major outliers appeared to be random with different participants exhibiting these extreme performance levels across measures with outliers sometimes appearing in the original baseline measures and other times in the follow-up measures. The lone exception to this summary of outliers was the measured gap-detection threshold at 1000 Hz. Here, four baseline and six follow-up measures were identified as major outliers and disregarded. Even here, however, 94 of 98 baseline and 92 of 98 follow-up 1000-Hz gap-detection thresholds were retained for subsequent analyses.

Three different approaches were used to examine the results from this longitudinal follow-up study. First, a series of paired-samples *t* tests were performed between baseline and 9-year follow-up measures. Second, correlations and slopes were calculated for the measures between baseline (T1) and follow-up (T2) intervals. Correlations were also used to examine the association between *rates* of sensory and cognitive decline over this 9-year period. Finally, the associations between baseline (T1) sensory performance and follow-up (T2) cognitive performance were examined via multiple-regression analyses to see if sensory function 9 years earlier (T1) predicted current (T2) cognitive function.

## Results and Discussion

### Group Comparisons Between Baseline (T1) and Follow-up (T2) Measures

**Figure 2.**
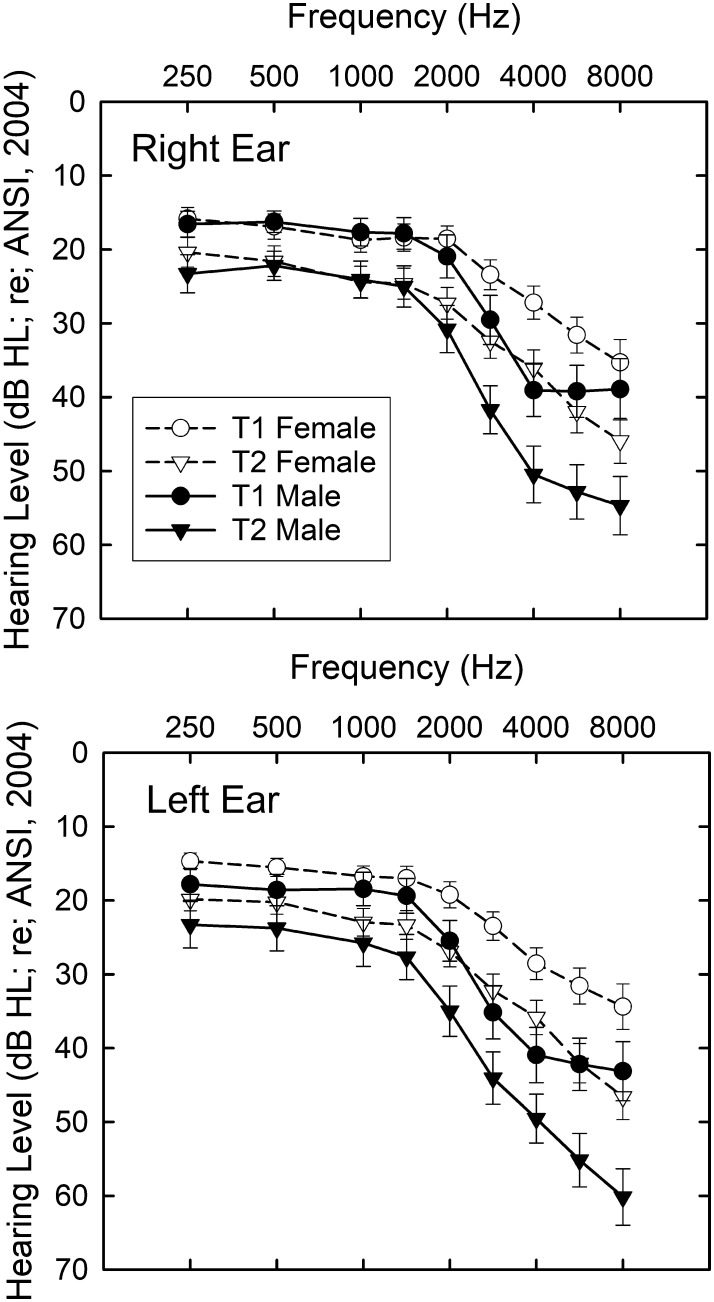
Means and standard errors of the air-conduction pure-tone audiometric thresholds at baseline (T1; circles) and at the 9-year follow-up (T2; triangles) for females (unfilled symbols) and males (filled symbols). Threshold data for the right ear are provided in the top panel and left ear in the bottom panel.

Figure 2 shows the means and standard errors for the audiograms at baseline (circles) and 9-year follow-up (triangles) for the right (top) and left (bottom) ears of the 98 participants. Results are shown separately for males (filled symbols) and females (unfilled symbols) in each panel. A mixed general linear model (GLM) analysis was performed with within-subject variables of test (T1, T2), ear (right, left), and frequency (250–8000 Hz) and a between-subjects variable of gender (male, female). Significant (*p* < .001) main effects of test, *F*(1, 96) = 209.2; frequency, *F*(8, 768) = 120.4; and gender, *F*(1, 96) = 4.0, *p* < .05, were observed, but not ear. The only significant interactions were between frequency and gender, *F*(8, 768) = 6.0, and test and frequency, *F*(8, 768) = 18.7, both of which are clearly visible in Figure 2. For a given test time (T1, T2), the difference between the hearing thresholds of males and females increases above 1500 Hz. In addition, for both genders, the difference in hearing thresholds from T1 to T2 increases with frequency. The observed changes in these clinically measured hearing thresholds are in line with other longitudinal reports for participants of similar ages (e.g., Gates & Cooper, 1991; Lee et al., 2005; Pearson et al., 1995; Wiley et al., 2008).

**Figure 3.**
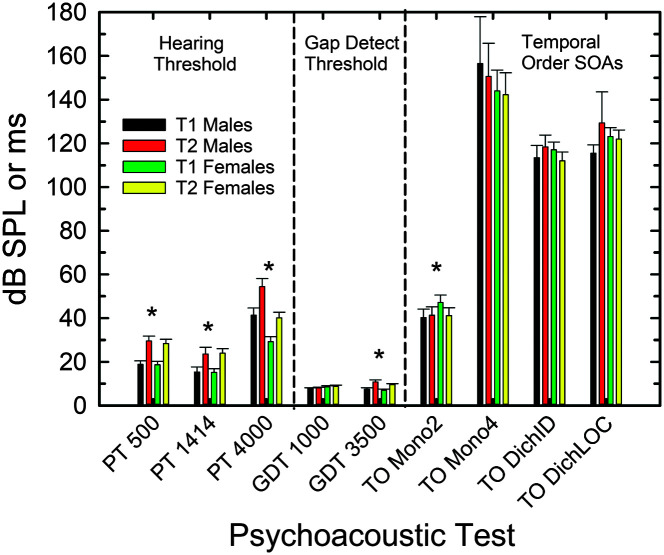
Means and standard errors for all auditory psychophysical measures at baseline for males (black bars) and females (green bars) as well as at follow-up 9 years later for males (red bars) and females (yellow bars). PT = pure-tone threshold in dB SPL; GDT = gap-detection threshold in ms; TO = temporal-order stimulus onset asynchrony (SOA) in ms; Mono = monaural temporal-order task, either 2-item or 4-item sequence; DichID = dichotic 2-item temporal-order task with identification; DichLOC = dichotic 2-item temporal-order task with location or ear response required. Asterisks mark the measures with significant effects of test (T1, T2) in generalized linear model analyses.

Figure 3 shows the means and standard errors for each of the nine auditory psychophysical measures. A mixed GLM analysis with within-subject variables of test (T1, T2) and frequency (500, 1414, 4000 Hz) and a between-subject factor of gender (female, male) was performed for the psychophysically measured thresholds (far left). A significant effect of test, *F*(1, 92) = 220.4, *p* < .001, was observed, and those differences over the 9-year interval found to be significant are marked by asterisks in this figure. The effects of frequency, *F*(2, 91) = 121.1, *p* <.001, as well as the interaction of test with frequency, *F*(2, 91) = 4.6, *p* < .05, and frequency with gender, *F*(2, 91) = 9.9, *p* < .001, were also significant but the main effect of gender was not significant, *F*(1, 92) = 1.3, *p* > .10. Except for the latter finding, these results are consistent with the clinical audiometric data in Figure 2 with the magnitudes of those changes also being similar to those measured clinically.

For the gap-detection thresholds (see Figure 3, center panel), another mixed GLM analysis was performed with within-subject variables of test (T1, T2), frequency (1000, 3500 Hz), and a between-subjects factor of gender (female, male). Significant effects were found for test, *F*(1, 83) = 74.8, *p* < .001, and the interactions of test with frequency, *F*(1, 83) = 91.7, *p* < .001, and gender with frequency, *F*(1, 83) = 8.0, *p* < .01. No other main effects or interactions were significant (*p* > .10). Regarding the effect of test, only those gap-detection thresholds for the noise band centered at 3500 Hz showed significant changes, with the T2 follow-up thresholds being 3.1 ms longer than those measured at the T1 baseline. Even though care was taken to ensure audibility of the stimuli, including the higher frequency noise band used in gap detection in this study, it remained possible that the threshold elevation in this same frequency region (4000 Hz) might underlie the effect of test at 3500 Hz. Threshold elevation is both a limiter of audibility and a marker for the severity of underlying cochlear pathology (Humes, 2007). Furthermore, because males had worse thresholds than females at 4000 Hz at T2, this may explain the interaction of frequency with gender noted above. To explore this further, the mixed GLM analysis was repeated with 4000-Hz threshold at T2 (T2pt4k) as a covariate. When doing so, the only significant effects that remained were the two- and three-way interactions with the T2 psychophysical threshold at 4000 Hz [test × T2pt4k, *F*(1, 81) = 4.4, *p* < .05; frequency × T2pt4k, *F*(1, 81) = 5.4, *p* < .05; and Test × Frequency × T2pt4k, *F*(1, 81) = 8.1, *p* < .01]. This suggests that there was no direct effect of advancing age (test), frequency, or gender on gap-detection thresholds but effects that were mediated by elevated thresholds in the region of the 3500-Hz gap-detection stimulus.

Finally, regarding the auditory temporal-order measures, shown in the right panel of Figure 3, a separate mixed GLM analysis was performed for each of the four measures of temporal-order identification performance with a within-subject factor of test (T1, T2) and a between-subjects factor of gender (female, male). Gender was not significant (*p* > .10) for any of these analyses, and the effects of test or the interaction of test with gender were found to be significant only for the monaural two-item temporal-order measure, test, *F*(1, 91) = 4.7, *p* < .05; and Test × Gender, *F*(1, 91) = 4.4, *p* < .05. The asterisk in the right panel of Figure 3 marks this lone significant effect of test and also illustrates that the effect of test was larger in females than males. Importantly, performance *improved* by 8.5 ms at follow-up compared to baseline in female participants.

In summary, of the nine psychophysical measures of auditory function completed here, four showed significant declines in performance over the 9-year interval. Three of the four measures showing significant declines were of hearing threshold, consistent with the significant declines observed for clinical measures of hearing loss shown previously in Figure 2. The only temporal-processing measure to show a significant decline over the 9-year interval was the gap-detection threshold at 3500 Hz, but this was largely explained by corresponding declines in hearing threshold at 4000 Hz. On the other hand, of the four auditory temporal-processing measures making use of complex stimuli (brief vowels) and a higher-level identification task, only one (monaural, two-item) showed a significant change and that was actually in a direction supporting *improved* performance over the 9-year interval, but only for female participants.

It should be noted that when one examines the two different sets of pure-tone thresholds for the right ear, from the audiogram and the psychophysical laboratory measures, there are some consistent differences. At 500 and 4000 Hz, the psychophysical thresholds measured with forced-choice procedures in the laboratory tend to be about 3 dB higher than the corresponding audiometric measures. Comparing the laboratory thresholds at 1400 Hz to the audiometric thresholds at 1500 Hz, there is better agreement at both the T1 baseline and T2 follow-up. Marshall and Jesteadt (1986) and Marshall (1991) found an average 6.5-dB difference, but with forced-choice methods targeting 70%–75% correct, as here, yielding *lower* thresholds than observed via audiometry. As will be seen below when discussing the correlations between T1 and T2, both the clinical and laboratory measures of hearing threshold were very reliable with T1-to-T2 correlations between .86 and .91 at each frequency in the same ear. The origins of this difference in threshold estimates between the methods are unclear, but the differences do appear to be stable over time. Also, when the correlations between the thresholds at each frequency were examined across methods at T1 and then again at T2, they were all strong and significant, ranging from .72 to .97. These correlations were lowest, however, for the 500-Hz frequency, 0.72 and 0.78 at T1 and T2, respectively; highest for the 4000-Hz frequency, 0.97 at both T1 and T2; and in between for the 1400/1500-Hz frequencies, 0.83 and 0.91 at T1 and T2, respectively. Thus, there are small (0–3 dB), but consistent, differences in pure-tone thresholds measured clinically and with forced-choice procedures in the laboratory with the poorest agreement between methods at 500 Hz at both T1 and T2.

**Figure 4.**
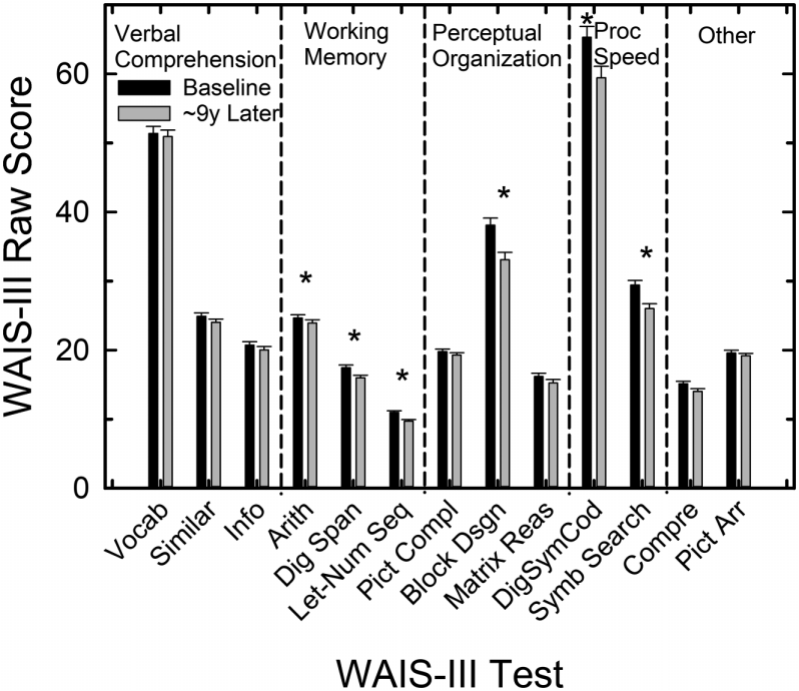
Means and standard errors for the raw Wechsler Adult Intelligence Scale–Third Edition (WAIS-III) scale scores at baseline (black bars) and follow-up 9 years later (gray bars). Asterisks mark significant differences between baseline and follow-up means from the generalized linear model analyses. The measures are grouped according to measurement type: verbal comprehension, working memory, perceptual organization, processing speed, or other.

Figure 4 shows the means and standard errors for the 98 older adults at T1 baseline (black bars) and T2 9-year follow-up (gray bars) for the 13 standard scales of the WAIS-III. Eleven of the 13 scales are used to generate index scores for four general cognitive abilities: verbal comprehension, working memory, perceptual organization, and processing speed. Each of the first four panels partitions the scale scores according to these indices with the two remaining scale scores, Comprehension and Picture Arrangement, shown at the far right (“Other”). Thirteen separate mixed GLM analyses were performed, one for each scale score shown in Figure 4, with a within-subject variable of test (T1, T2) and a between-subject variable of gender (female, male). Given the number of analyses performed on the WAIS-III measures, the criterion *p* value for significance was Bonferroni adjusted (*p* = .05/13 = .0038). Gender was found to have a significant main effect (males with higher scores) only for the arithmetic scale, *F*(1, 96) = 24.7, and the information scale, *F*(1, 96) = 9.4, and no interactions between test and gender were significant. As a result, the scores in Figure 4 are not shown separately for males and females.

The effects of test were found to be significant for the scales of digit–symbol coding, block design, arithmetic, digit span, symbol search, and letter–number sequence, all *F*(1, 96) > 11.7. Each of these significant effects of test has been marked with an asterisk in Figure 4. All three measures of working memory and both measures of processing speed show significant declines over the 9 years. In addition, one of the three scores comprising the perceptual organization index, block design, shows a significant decline over the 9-year interval between T1 baseline and T2 follow-up. Recall that the scores in Figure 4 are all raw scores for these scales, not age-adjusted scores. As a result, significant declines are expected over the 9-year interval in processing-based measures, such as working memory and processing speed, but not in product-based measures like verbal comprehension and perceptual organization (e.g., Salthouse, 2010a).

In the literature on aging, especially age-related changes in cognition, there have been debates as to whether cross-sectional data provide an accurate depiction of life span changes (e.g., Salthouse, 2010b, 2011; Schaie, 2005). The primary weakness of cross-sectional designs lies in the use of different cohorts for each age along the life span and that other differences, such as educational or socioeconomic generational differences, may contribute to the observed “age-related” changes. On the other hand, as noted previously, longitudinal approaches have the potential to confound practice or learning effects with age-related changes due to the repeated measurements within the same individuals. To compare the prior cross-sectional data from the baseline measures to the present longitudinal data, the 245 adults, including young adults, tested at T1 baseline, ranging in age from 18 through 85 years, were divided into age deciles with about 25 individuals per decile. Given the smaller total *N* for the T2 9–year follow-up data (*N* = 98), that group was divided into age quartiles again with about 25 individuals per quartile.

**Figure 5.**
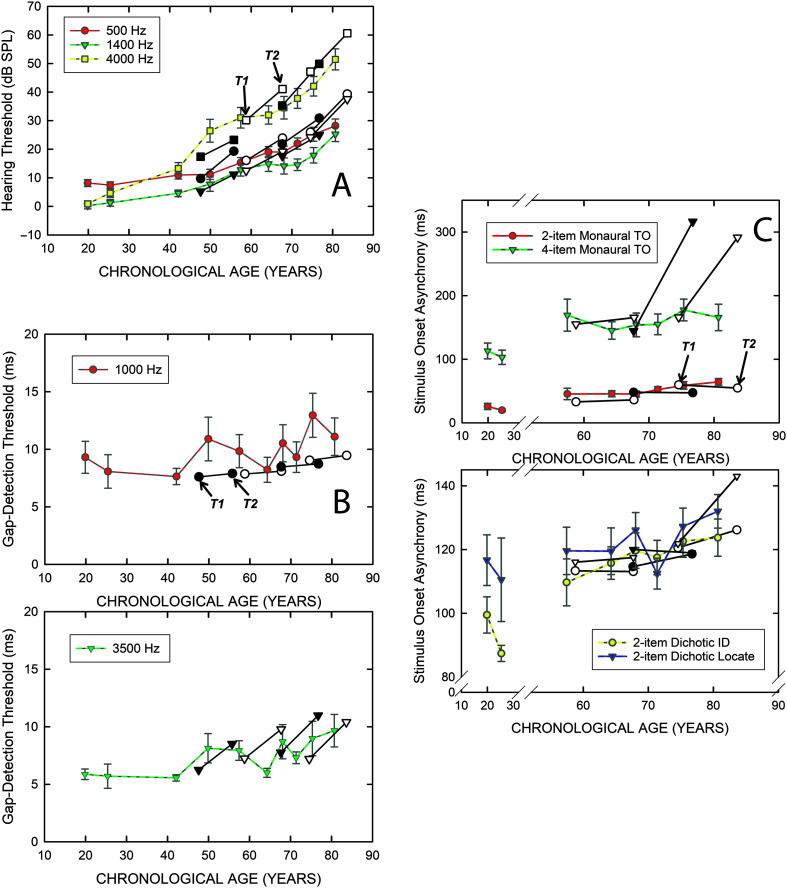
Comparison of the means and standard errors for all auditory measures between each T1 age decile of the original baseline cross-sectional data set of Humes, Busey, et al. (2013; *N* = 245), colored symbols and lines, and the means and standard errors for the longitudinal data (T1, T2, black and white paired symbols connected by solid black lines) for the T2 age quartiles (*N* = 98). Each pair of black or white symbols connected by a solid black line shows the change from T1 to T2 for a specific T2 age quartile. (A) psychophysical auditory thresholds. (B) gap-detection thresholds for 1000 Hz (top) and 3500 Hz (bottom). (C) stimulus-onset asynchronies for monaural (top) and dichotic (bottom) temporal-order identification tasks.

Figure 5 shows the comparisons between the cross-sectional data (yellow, blue, red, or green symbols with error bars) and the longitudinal data (black or white symbols) for all the psychophysical measures. The longitudinal data are shown as two data points connected by a solid black line for each age quartile, with each quartile having a different age at baseline (T1) and 9-year follow-up (T2). An example T1–T2 quartile data pair showing the longitudinal progression in performance for that quartile has been identified by an arrow in each panel of Figure 5. Hearing thresholds are at the top left (A), gap-detection thresholds in the middle and bottom left panels (B), and auditory temporal-order SOAs in the two right panels (C). Notice that there is a break in the *x*-axis in Panel C for the temporal-order identification SOAs due to missing data among the 40- to 50-year-olds for either the baseline or follow-up intervals, which left only about two thirds of the subjects with complete data in this age range for these measures. There is good agreement between the cross-sectional (colored symbols) and longitudinal data (black or white symbols) for the auditory measures in Figure 5 for all but one measure: monaural four-item temporal-order identification (C, top). The longitudinal data for the two older quartiles on the monaural four-item temporal-order task (Mono4) suggest more rapid declines (*increases* in SOA) with age than do the cross-sectional data over those same age ranges. At both the baseline (T1) and follow-up (T2) intervals, the 4-item temporal-order task had the most frequent occurrence of “could not test” entries. Such entries were generated whenever the maximum SOA was exceeded and valid thresholds could not be obtained. It is most likely that the true SOA was larger, but because the SOA exceeded the maximum limits and its precise value was unknown, such results were designated as “missing” here. If, however, it is correct to assume that the true values were higher than could be measured, then these missing values could be replaced by arbitrary high SOAs with the *medians* at T1 and T2, rather than the means, providing a better indication of the change in SOA for the four-item temporal-order task. For the second, third, and fourth age quartiles shown in the top panel of Figure 5C, the difference in median SOAs calculated in this way (replacing all missing values with an SOA of 2,000 ms) from T1 to T2 was +7.3, −19.5, and +26.7 ms, respectively. These changes in SOA for the Mono4 condition are much smaller than the 100+ ms shown for the third and fourth age quartiles in Figure 5C and likely better reflect the true longitudinal changes for each quartile on this task. Thus, with this adjustment, there is good agreement between the cross-sectional and longitudinal measures for *all* the auditory psychophysical tasks shown in Figure 5.

**Figure 6.**
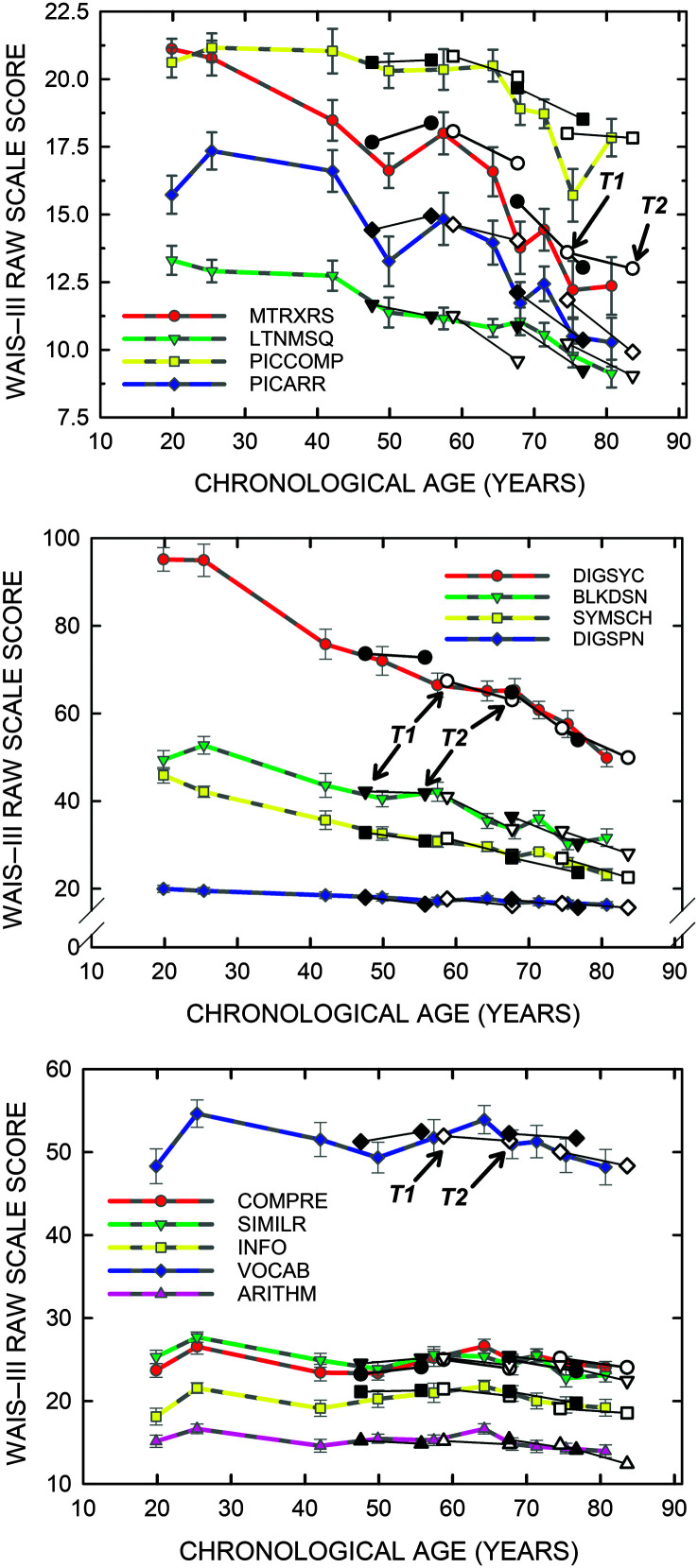
Comparison of the means and standard errors for all Wechsler Adult Intelligence Scale–Third Edition (WAIS-III) raw scale scores for each T1 age decile (*N* = 245) from the prior cross-sectional data from Humes, Busey, et al. (2013), colored symbols and lines, to the means and standard errors for the longitudinal data (T1, T2, black and white symbols) for the T2 age quartiles (*N* = 98). Each connected pair of black or white symbols shows the change from T1 to T2 for a specific T2 age quartile. Top two panels illustrate results for several of the process-based tests, sorted by size for clarity of depiction, whereas the bottom panel includes the product-based tests.

Figure 6 shows a comparable analysis of the WAIS-III cognitive scale scores. Good agreement is observed between the cross-sectional data and the longitudinal data for all 13 WAIS-III scale scores. The top two panels show scale scores that steadily decline with age and are considered to be process-related cognitive measures, whereas the bottom panel shows scale scores that are considered to be product-related and more stable over the entire adult life span until 75–80 years of age (Salthouse, 2010a). The absence of learning effects in these data is consistent with the recommendation of Salthouse (2011) that the T1–T2 intervals should be at least 8 years to avoid this confound in longitudinal studies of cognition.

In summary, although there were several significant changes for the group means from T1 to T2 for the 98 older adults (see Figures 3 and 4), when the average performance at the T1 and T2 measurement points is compared to the cross-sectional data at comparable age ranges (see Figures 5 and 6), there is very good agreement between the cross-sectional trends and the longitudinal data. This supports the arguments of Salthouse (2010a, 2011) regarding the validity of cross-sectional data as a depiction of average age-related changes in function over the adult life span, extended here to measures of auditory function as well as cognitive function. Importantly, the agreement observed here between the cross-sectional and longitudinal average data likely hinges on the relatively long interval, about 9 years, between T1 baseline and T2 follow-up for the longitudinal measures. Use of shorter T1–T2 intervals may not have yielded the same agreement between the average data for the two approaches (Salthouse, 2011).

Although both approaches may yield similar trends in the average data, an added advantage of the longitudinal approach is the ability to look for correlations among measures within individuals and *over time* (Evans, 1978; Schaie, 1983, 2005). It is from such correlations and measures of change over time within the same individuals that theories such as the deprivation and common-cause theories of sensory-cognitive interactions in aging have emerged (Humes & Young, 2016; Lindenberger & Baltes, 1994; Pronk et al., 2019; Schneider & Pichora-Fuller, 2000; Wayne & Johnsrude, 2015). Such correlations and measures of change within the same individuals over time are the focus of the remaining analyses of the results from this study.

### Correlations and Slopes Relating T1 Baseline and T2 Follow-Up Measures

**Figure 7.**
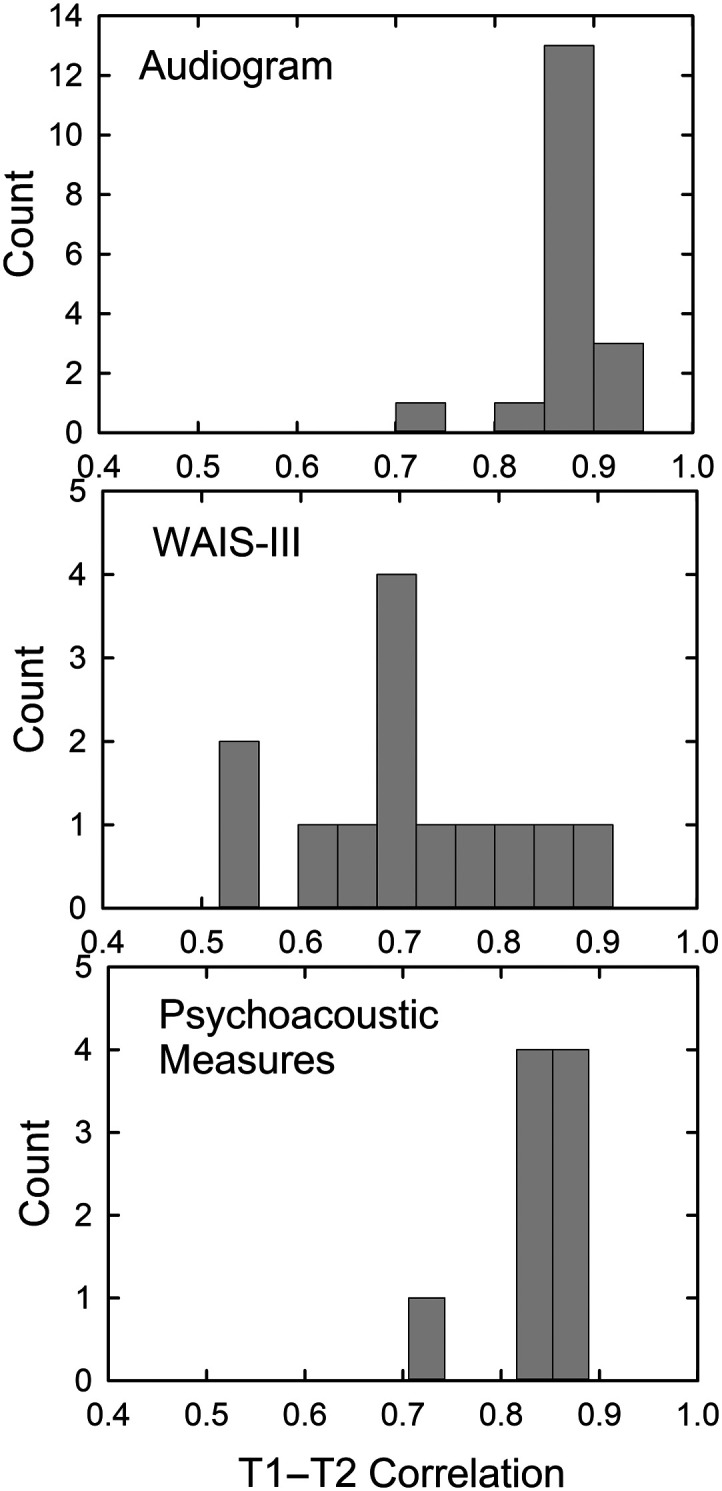
Histograms of correlations from baseline (T1) to 9-year follow-up (T2) for audiometric thresholds (top), Wechsler Adult Intelligence Scale–Third Edition (WAIS-III) raw scale scores (middle), and psychoacoustic measures of hearing threshold, gap-detection, and temporal-order identification (bottom).

Figure 7 shows histograms of the Pearson *r* correlation coefficients between T1 baseline and T2 9-year follow-up measures for the audiogram (top), WAIS-III (middle), and psychoacoustic measures (bottom). As can be seen, all these measures were moderately to highly correlated and all are statistically significant (*p* < .05). The correlations suggest a relatively consistent ordering of participants in terms of their auditory and cognitive performance across the 9-year interval, a little more so for the auditory measures than for the cognitive measures.

**Figure 8.**
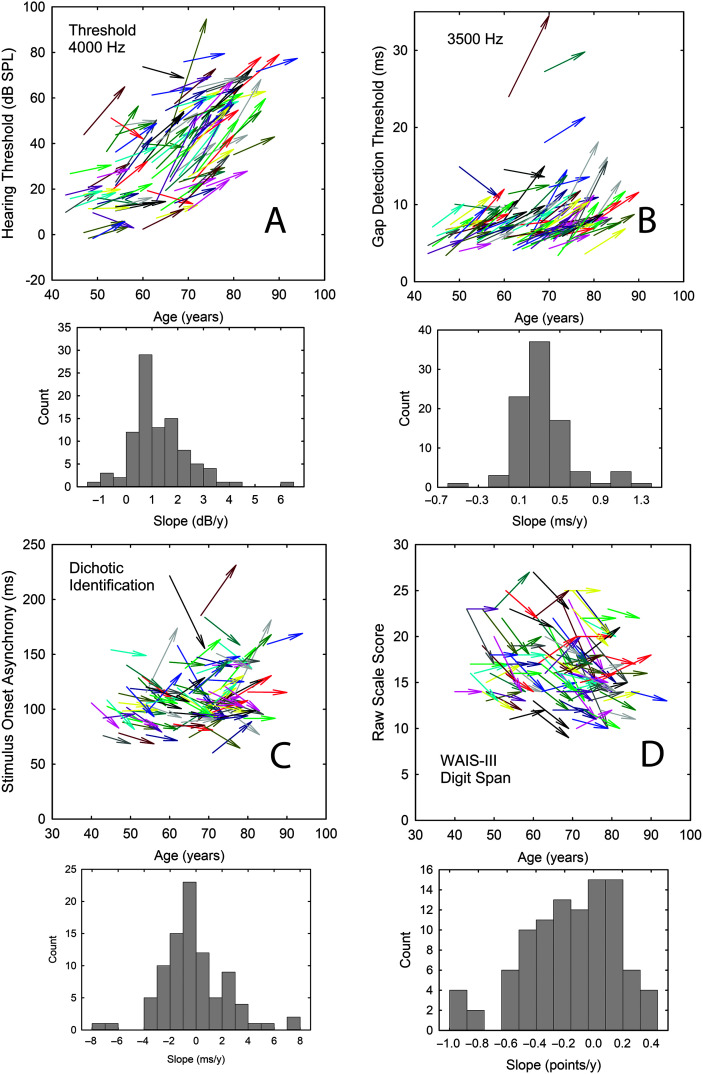
Examples of individual longitudinal data illustrating change from baseline (T1) to follow-up (T2) via individual colored arrows top panels of each pair and ensuing histogram of resulting slopes for that same measure (gray bars in bottom panel of each pair). Representative data are shown for one case of each of the three types of auditory psychophysical measures (A: pure-tone hearing threshold at 4000 Hz; B: gap-detection threshold at 3500 Hz; C: temporal order, dichotic identification) and Wechsler Adult Intelligence Scale–Third Edition (WAIS-III)\ raw scores (D: digit span).

The magnitudes of the correlations are sufficient to support the use of 2-point linear slope estimates: slope = (Performance at T2 − Performance at T1)/(T2 − T1 interval in years). For all the auditory measures, a higher threshold value at T2, whether in dB or ms, reflects poorer performance and such a decline in performance over the 9-year interval would be reflected in a positive slope. For the cognitive measures, however, poorer performance is reflected in lower WAIS-III scores and age-related declines would yield negative slopes in this case. Figure 8 illustrates several representative examples of the slopes for psychophysical measures of hearing threshold (A; 4000 Hz), gap-detection threshold (B; 3500 Hz), temporal-order identification SOA thresholds (C; DichID), and cognitive WAIS-III raw scale scores (D; Digit Span). The start and end points of each arrow in the upper panels represent the T1 and T2 values for each of the 98 subjects (major outliers deleted), and the histograms at the bottom of each panel show the relative distribution of the slopes calculated as described above. These data illustrate that, although there are central tendencies for the slope estimates for the group, there are reasonably large individual differences in slopes. Individual differences in the rate of sensory change over the T1–T2 interval may be associated with corresponding changes in cognitive function over the same interval, and this will be the focus of subsequent sections of this report.

**Figure 9.**
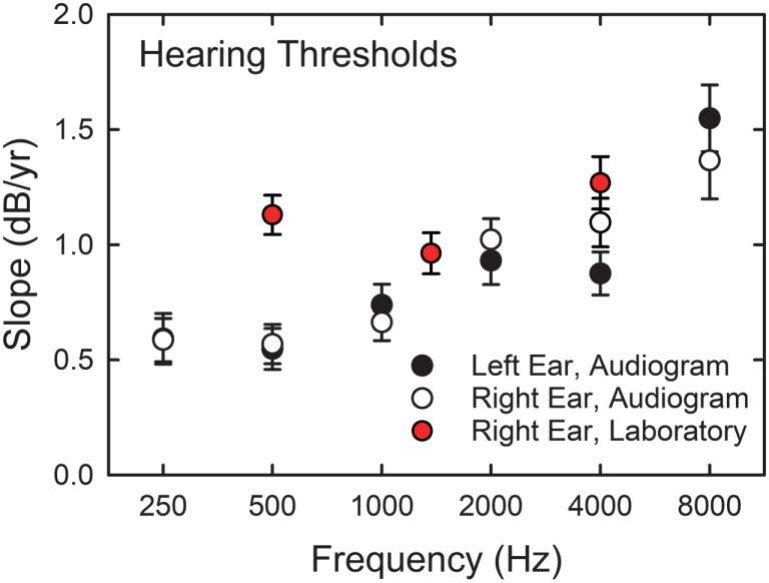
Slopes for the changes in hearing threshold from T1 baseline to T2 follow-up 9 years later with clinically measured pure-tone thresholds shown as black (left) or white (right) circles and psychophysical laboratory thresholds shown as red circles (right ear only). Error bars represent ±1 *SE*.

Figure 9 shows the observed slopes, means, and standard errors for clinical (black and white circles) and psychophysical (red circles) measures of hearing threshold. There are more longitudinal studies of audiometric data like these than any other auditory measure, but the details regarding the rates of decline and the effects of frequency vary considerably among the prior studies (Lee et al., 2005; Pearson et al., 1995; Wiley et al., 2008). In general, however, for the age range spanned in this study, these studies show average rates of decline in hearing sensitivity of about 1 dB/year with varying effects of frequency, age, and gender across studies. For the audiometric data in Figure 9, a slope of 1 dB/year would be a representative value. There was a significant effect of frequency for both the right, *F*(5, 485) = 13.25, *p* < .01, and left, *F*(5, 485) = 17.92, *p* < .01, ear slopes. For the right ear, post hoc Bonferroni-adjusted *t* tests indicated that the audiometric slopes at 250, 500, and 1000 Hz were each significantly (*p* < .05) lower than those at 2000, 4000, and 8000 Hz, but there were no significant differences within each of these three-frequency sets. For the left ear, the slope at 8000 Hz was significantly greater (*p* < .05) than that at all other frequencies and the slope at 500 Hz was significantly lower than all other frequencies except for 250 Hz. No other significant differences in slope were observed for the left ear.

For the psychophysically measured pure-tone thresholds (see Figure 9, red symbols), a general average value for the slopes would again be about 1 dB/year. The effect of frequency was not significant, *F*(2, 186) = 3.48, *p* > .01. Whereas the slopes at the two higher frequencies, 1400 and 4000 Hz, generally agree with those from the audiometric data, the slope at 500 Hz is nearly twice that observed in the audiograms for that same frequency. The reasons for this difference between the slopes for the psychophysically measured threshold and the audiometric threshold at 500 Hz are not clear.

The effects of participant gender and age on the slopes in Figure 9 were also examined. No significant (*p* > .05) effects of gender were observed for the hearing-threshold slopes for either the clinical or psychophysical measures and at any of the frequencies. Age effects on slopes were examined using the same age quartiles described previously with about 25 participants per quartile. The four quartiles ranged in age at baseline from 40–53, 54–64, 65–70, and 71–85 years with mean baseline ages of 47.6, 58.8, 67.6, and 74.6 years, respectively. As noted above, the slopes were defined as threshold at T2 follow-up minus threshold at T1 baseline, divided by the interval between these two measurement points. There was a small, but significant (*p* < .05), difference in the baseline-to-follow-up interval across the four age quartiles such that the mean interval for the youngest age quartile was lower (8.2 years) than that of the other three quartiles (8.9, 9.1, and 9.0 years). As a result, the GLM analyses examining the effects of age quartile on the slopes for pure-tone thresholds were performed with the T2–T1 interval as a covariate. For the audiometric thresholds, only the slopes at 8000 Hz in each ear failed to show significant differences across age quartiles (*p* > .05). There are 30 possible comparisons per ear remaining to evaluate, six pairwise age–quartile comparisons at each of the five remaining frequencies, 250–4000 Hz. Eleven of these 30 paired comparisons in the right ear and nine of the 30 paired comparisons in the left ear were significant (*p* < .05). In both ears, all but one of the significant paired comparisons involved the slope of the oldest quartile being significantly greater than that of a younger age quartile, and, 5 times for each ear, it was specifically that slope in the oldest quartile being steeper than that of the youngest quartile. Of the 10 significant age–quartile differences in the right ear involving the oldest quartile, eight occurred at 250, 500, or 1000 Hz. Similarly, of the eight significant age–quartile differences in the left ear involving the oldest quartile, five occurred at these same three lower frequencies. In summary, the progression of hearing loss tended to be steeper for the oldest quartile, people who were in their mid-70s at baseline and aged to their mid-80s at follow-up, and this was most often observed at frequencies of 250, 500, or 1000 Hz. Otherwise, about two thirds of the paired comparisons among age quartiles in each ear were nonsignificant, indicating that there were no effects of baseline age on the rate of the progression of hearing loss.

For the psychophysical laboratory measures of threshold, the GLM analyses examining the effects of age quartile on the slopes for pure-tone thresholds were again performed with the T2–T1 interval as a covariate. Significant effects (*p* < .05) of age quartile were observed at 1400 Hz and 4000 Hz, but not at 500 Hz. At 1400 Hz, the oldest group differed significantly from each of the three younger quartiles; whereas at 4000 Hz, it was only the third quartile that differed from the youngest quartile.

As noted, the prior longitudinal studies of hearing thresholds across the adult life span have made use of audiometric data and expressed the changes in hearing as slopes in dB/year. For the cognitive measures, on the other hand, the most appropriate way to express the slopes is in *z*-transformed rates with the *z* scores for baseline and follow-up calculated relative to the baseline's mean and standard deviation (Salthouse, 2010a, 2010b, 2011). This is common practice in cognitive studies of aging because the range of raw scores for each cognitive measure, like those in the WAIS-III, can vary over an order of magnitude, as illustrated previously in Figure 6. Given that the denominator of the slope calculation is basically a constant, averaging about 9 years here, steeper slopes will be obtained for comparable proportional declines when the raw scores are of greater magnitude. *z* scores offer a way to normalize the range of raw scores across measures, and, by tying both the T1 baseline and T2 follow-up z scores to the mean and standard deviation at T1 baseline, the slope represents the change relative to baseline (Salthouse, 2010a, 2011). Because the measures of temporal processing ranged from 5–10 ms for gap-detection to greater than 100 ms for the dichotic temporal-order measures, as shown previously in Figure 5, *z*-transformed slopes were calculated in the same manner for the temporal-processing measures. For completeness and to facilitate subsequent correlational analyses between sensory and cognitive changes, these same *z* score–based slopes were calculated for the three psychophysically measured hearing thresholds.

The raw and *z* score–based slopes are provided in Table 1. Each was evaluated with a *t* test to determine if it differed significantly from a slope of zero. Those significantly (*p* < .01) different from a value of 0 are marked with an asterisk. For the auditory measures, hearing thresholds worsened significantly at all three frequencies, as did gap detection at 3500 Hz only. For the temporal-order identification measures, only the monaural two-item task had a slope that differed significantly from 0, and this was in the direction of improved performance at follow-up. These results are consistent with the results presented previously (see Figure 3) for the auditory measures. For the cognitive measures from the WAIS III, seven scales show slopes that differ significantly from 0: digit–symbol coding, block design, arithmetic, digit span, information, symbol search, and letter–number sequencing. Six of these same seven WAIS-III scales showed significant differences between mean scores at baseline and follow-up via paired-samples *t* tests, as shown previously in Figure 4.

**Table 1.** Means and standard deviations for the raw and *z*-transformed (re: T1) slopes for change from T1 to T2.

Measure	*N*	Mean raw	*SD* raw	Mean *z*	*SD* *z*
PT 500 Hz	95	1.14	.83	.10*	.07
PT 1400 Hz	96	.96	.88	.07*	.06
PT 4000 Hz	95	1.27	1.11	.07*	.06
GDT 1000 Hz	88	.04	.22	.01	.06
GDT 3500 Hz	91	.34	.27	.08*	.07
TO Mono2	93	−.58	1.72	−.02*	.07
TO Mono4	72	−.41	4.22	−.01	.05
TO DichID	89	−.18	2.45	−.01	.08
TO DichLOC	85	.02	1.82	.00	.06
WAIS-III PicComp	95	−.06	.33	−.02	.09
WAIS-III Vocab	98	−.05	.63	−.01	.06
WAIS-III DigSyC	98	−.64	1.09	−.04*	.07
WAIS-III Similar	98	−.09	.43	−.02	.09
WAIS- III BlkDsgn	98	−.55	.76	−.05*	.08
WAIS-III Arith	98	−.12	.35	−.04*	.10
WAIS-III MtrxRs	98	−.10	.43	−.02	.09
WAIS-III DigSpn	98	−.17	.31	−.04*	.08
WAIS-III Info	98	−.08	.23	−.02*	.05
WAIS-III PicArr	98	−.11	.47	−.03	.11
WAIS-III Compreh	98	−.09	.42	−.02	.10
WAIS-III SymSch	97	−.39	.60	−.06*	.09
WAIS-III LtNmSq	98	−.14	.24	−.07*	.11

*Note.* Sample sizes for each measure are also shown, and means that significantly (*p* < .01, unadjusted) differed from zero are marked by an asterisk. For the raw slope, the pure-tone threshold slopes are dB/year, the temporal-processing slopes are ms/year, and the WAIS-III slopes are scale points/year. PT = pure-tone threshold; GDT = gap-detection threshold; TO = temporal-order threshold; Mono2 = monaural two-item identification; Mono4 = monaural four-item identification; DichID = dichotic two-item vowel identification; DichLOC =dichotic two-item ear or location identification; WAIS-III = Wechsler Adult Intelligence Scale–Third Edition.

Associations were next examined between the *z*-normalized slopes for the auditory measures and the cognitive measures. Table 2 shows the partial correlations between the auditory and cognitive measures when controlling for both baseline age and the interval between baseline and follow-up measures. A total of 10 correlations were found to be statistically significant (*p* < .05 or *p* < .01) as indicated by bold font and asterisks in Table 2. Eight of the 10 are in a direction consistent with an association between declining auditory function and declining cognitive function. Under this assumed association, the correlations would be expected to be negative reflecting increasing auditory thresholds, in dB or ms, and decreasing WAIS-III scale scores. For the two significant positive correlations, one involves the Picture Arrangement subtest of the WAIS-III, which has been dropped from the subsequent edition of the WAIS, WAIS-IV (Wechsler, 2008), due to its poor reliability and other concerns. The remaining significant positive correlation is between the gap-detection threshold at 3500 Hz and WAIS-III Block Design performance, a timed measure of visual–spatial organization. There is no obvious explanation for this association. The eight remaining statistically significant partial correlations between auditory and cognitive function range in magnitude from −.21 to −.32, all relatively weak. In addition, it is noteworthy that six of the eight significant negative correlations involve one of the dichotic temporal-order identification tasks. It should be noted that the pattern and magnitude of partial correlations in Table 2 were very similar to those observed for correlations calculated without controlling for age or T2–T1 interval. For these standard zero-order Pearson *r* correlations, four additional correlations were found to be significant and three of these involved the dichotic measures.

**Table 2.** Partial correlations between *z*-transformed slopes of the sensory measures (columns) and cognitive measures (rows).

Measure	PT 500	PT 1400	PT 4000	GD 1000	GD 3500	Mono2	DichID	DichLOC
PicComp	.138	.200	.137	.074	.130	.102	−.054	.045
Vocab	−.114	−.050	−.158	.007	.160	−.207*	−.295**	−.205
DigSymC	.077	.030	−.035	.211	.140	−.033	−.134	−.082
Similar	−.129	−.194	−.067	−.017	.034	−.115	−.221*	−.060
BlkDsgn	.029	−.044	−.157	.156	.219*	−.135	−.161	−.150
Arith	−.100	−.028	−.059	−.004	−.033	−.061	−.321**	−.072
MtrxRs	.037	.076	−.094	.036	.176	−.206	−.250*	−.291**
DigSpn	−.011	−.131	.025	−.029	.083	−.170	−.089	.018
Info	.065	.098	−.124	−.157	−.073	−.143	−.063	−.129
PicArr	.095	.318**	.113	.008	.183	−.160	−.063	−.153
Compreh	.103	−.049	−.089	−.078	.083	−.077	−.081	−.095
SymSrch	−.022	.130	.068	−.040	−.072	−.114	−.180	−.235*
LtNmSeq	−.083	−.039	−.247*	.038	−.051	−.039	−.144	.110

*Note.* Covariates were age at baseline and length of the interval from baseline (T1) to follow-up (T2). The four-item monaural temporal-order measures were excluded due to the higher percentage of missing data for this measure. Significant correlations are in bold and marked with one (*p* < .05) or two (*p* < .01) asterisks. PT = pure-tone threshold; GD = gap-detection threshold; Mono2 = monaural two-item identification; Mono4 = monaural four-item identification; DichID = dichotic two-item vowel identification; DichLOC = dichotic two-item ear or location identification.

The magnitudes of the partial correlations in Table 2 indicate that about 4%–10% of the variance in the rate of cognitive decline can be explained by the rate of auditory decline over the same period. By controlling for age and T2–T1 interval, moreover, this is an age-independent association among these auditory and cognitive rates of decline. Although this represents a small proportion of variance explained, these correlations are in line with prior longitudinal studies examining the association between sensory and cognitive decline in older adults (Lindenberger & Baltes, 1994; Lin, Ferrucci, et al., 2011; Lin, Metter, et al., 2011; Lin et al., 2013; Livingston et al., 2017; Loughrey et al., 2018). For example, the systematic review conducted by Lancet Commission on Dementia Prevention, Intervention & Care (Livingston et al., 2017) identified hearing loss as the top modifiable factor predicting incident dementia accounting for as much as 9% of the variance. The systematic review by Loughrey et al. (2018) is even more relevant as they examined the associations between hearing loss and cognitive function among healthy aging adults. They reported Pearson *r* correlations between hearing loss and cognitive measures, like those of the WAIS-III used here, for 26 cross-sectional studies that ranged from −.08 to −.18 with a mean correlation of *r* = −.12. Thus, hearing loss accounted for a little over 1% of the variance in cognitive measures among healthy aging adults, with a similar range of correlations reported for the nine longitudinal studies included in the review by Loughrey et al. (2018). The 6% variance explained by the 4000-Hz pure-tone threshold measured in the laboratory in this study is of similar magnitude to the 1%–9% noted in these recent systematic reviews.

The strongest associations between auditory and cognitive rates of decline among the older adults in this study, however, were not pure-tone thresholds, the sole auditory measure evaluated in the prior literature, but the dichotic temporal-order measures. The slope for the decline in dichotic temporal-order task requiring identification of the vowel sequence across the two ears (DichID), controlling for T1 age (and T2–T1 interval), explained 4.9%–10.3% of the variance in cognitive-decline slopes over the same period. Temporal-order identification also showed the strongest link to cognitive function in structural-equation modeling of the original cross-sectional data from 245 young, middle-aged, and older adults (Danielsson et al., 2019). For dichotic temporal-order judgments requiring only the identification of the ear stimulated first (DichLOC), the correlations in Table 2 indicate that 5.5%–8.5% of the variance in cognitive-decline slopes was explained by the rates of change for this auditory measure. It is also noteworthy that the significant negative correlations with the rates of decline in dichotic auditory measures cut across several different cognitive domains including verbal comprehension (Vocabulary, Similarities), working memory (Arithmetic), Perceptual Organization (Matrix Reasoning), and processing speed (Symbol Search). Gates et al. (2008, 2011) have suggested an association between poor dichotic listening performance and cognitive impairment. Here, we observed an association between rates of decline in dichotic performance and cognitive function among healthy older adults free from cognitive impairment at baseline (based on MMSE scores at T1 > 25).

It is perhaps not too surprising that the strength of the associations between hearing and cognition are greater for the dichotic measures used here rather than the measures of hearing loss or gap-detection threshold. Dichotic listening has long been recognized as having at least two components: a cognitive attentional component and an auditory-processing brainstem or cortical component (e.g., Bronkhorst, 2000, 2015, Cherry, 1953). The relative contributions of peripheral auditory processing and higher-level auditory and cognitive processing change as the complexity of the stimuli and listener's task change (Bronkhorst, 2015). Here, brief vowel stimuli, easily identified in isolation, were strung together in rapid sequences and, for three of the four conditions, the identification of each vowel in that sequence (from the set of four possible vowels) was required. The stimuli are relatively complex compared to the pure tones or burst of noise used in the other auditory measures examined here. Furthermore, the task also is more complex than simple detection or discrimination of sounds as in the other auditory tasks used here. Of the two dichotic tasks, DichID and DichLOC, the measure with the more complex response task, DichID, had four significant correlations with cognitive measures, whereas the simpler task of just identifying the ear stimulated first (DichLOC) was significantly correlated with cognitive function in only two cases. Note in Table 2 that only one correlation using the same stimuli and response task, but with the stimuli always delivered to the same ear (Mono2), showed a significant association with cognitive function. Is this because of differences in monaural versus binaural auditory processing or attentional differences? For the DichID task, the order of stimulus presentation to each ear was random with the first vowel presented to the right ear first half of the time. For the Mono2 task, both vowels were presented to the right ear all the time. That is, no shifting of attention from ear to ear or uncertainty of stimulus location from trial to trial was involved and perhaps this ear-uncertainty factor underlies the more frequent associations between DichID and cognition than between Mono2 and cognition. Fogerty et al. (2010) included a control experiment in which Mono2 measures were obtained but the ear stimulated varied randomly from trial to trial. The uncertainty of stimulus ear had no significant effect on Mono2 performance. So, it appears that it would be either binaural processing, the process of switching attention from ear to ear during the stimulus presentation, or both that may underlie the more frequent correlations between DichID and cognitive function noted in Table 2.

### Association between Baseline (T1) Sensory Function and Current (T2) Cognitive Function

In the prior two sections, the focus was on the *changes* within the cohort of older adults over the 9-year time span, whether the group data were analyzed via paired-samples *t* tests or the individual data were analyzed via correlations between T1 and T2 and the slopes from T1 to T2. Here, the question addressed is: Is current cognitive function at T2 related to auditory function measured 9 years earlier at T1? As noted previously, some models of the association between sensory and cognitive decline over the adult life span, such as the deprivation model, suggest that sensory decline precedes cognitive decline (Humes & Young, 2016; Lindenberger & Baltes, 1994; Pronk et al., 2019; Schneider & Pichora-Fuller, 2000; Wayne & Johnsrude, 2015).

Prior to examining the associations between baseline (T1) auditory measures and subsequent follow-up (T2) cognitive measures, the 13 WAIS-III scale scores obtained at follow-up were subjected to principal-components factor analysis (Gorsuch, 1983) for data reduction. A good fit was obtained with the Kaiser-Meyer-Olkin (KMO) measure of sampling adequacy = 0.86, all communalities ≥ 0.56, and 67.1% of the variance explained by three orthogonal (varimax rotation) components. The three factors were easily identified as a Processing Speed/Perceptual Organization (PSPO) factor, a Verbal Comprehension factor, and a Working Memory factor. Next, the auditory data from T1 were subjected to the same principal-components factor analyses with all audiometric thresholds and psychophysical measures, except for the monaural four-item temporal-order SOAs (Mono4), which were eliminated due to the high percentage of missing data. A good fit was once again obtained with the KMO sampling adequacy = 0.88, all communalities ≥ 0.60, and 80.8% of the variance explained by five orthogonal (varimax rotation) components: hearing loss at and above 2000 Hz bilaterally, hearing loss below 2000 Hz in the right ear, hearing loss below 2000 Hz in the left ear, gap detection, and temporal-order identification. For the psychophysical measures, there were some scattered missing values, with the worst case being eight of 98 values missing for the DichLOC temporal-order measure and the remaining seven measures having zero to four missing values. The principal components factor analysis of these measures was run both with list-wise deletion of missing data (*N* = 86), as well as with replacement of missing values by the means for that measure (*N* = 98), both yielding the same results (five orthogonal components with the same interpretation accounting for 81.6% and 80.8% of the variance with nearly identical KMO values and communalities). The factor scores for the analysis using the replacement of missing data with mean values were the factor scores retained for subsequent regression analyses.

It is also noteworthy that separate principal components emerged from this analysis of the auditory measures for hearing loss (three principal components), gap detection, and temporal-order identification. Importantly, hearing loss was a separate factor that emerged rather than one common to all measures in part because great care was taken to minimize the impact of hearing loss on the other auditory measures by selecting relatively high presentation levels and minimizing spectral overlap of the stimuli with the expected region of hearing loss. The emergence of three separate auditory factors in this analysis confirms the independence of each factor. For oblique rotation of the three components, which unlike orthogonal rotation allows for correlation among the rotated components, the correlations among the three components were all negligible, *r* = .12, .21, and .39, further supporting the independence of these three auditory factors.

Next, three separate multiple linear regression analyses were performed, one for each of the three T2 WAIS factors, using age and the five orthogonal auditory factor scores as the predictor independent variables. Because the factor scores for the WAIS and auditory measures were normalized to have a mean of 0 and a standard deviation of 1, the age variable was converted to *z*-scores to give it the same mean and standard deviation. For all linear multiple-regression analyses, collinearity diagnostics indicated that collinearity among the independent variables was not a concern (variance inflation factor [VIF] < 1.7, condition index between 1.0 and 2.1).

The results of the three regression analyses between baseline T1 auditory factor scores and follow-up T2 WAIS-III factor scores are provided in Table 3. For the regression analysis of the WAIS PSPO factor score, shown in the upper portion of Table 3, the regression equation was significant, *F*(6, 91) = 11.3, *p* < .001, and explained 39.0% of the variance (adjusted *r^2^*). The standardized beta coefficients are shown in Table 3, together with the *t* test and various correlations. Two significant T1 predictors of the WAIS-III PSPO factor score were identified, age and temporal-order performance, with age as the primary factor, as reflected by the partial and part (semipartial) correlations in Table 3. Based on the partial and part correlations, temporal-order performance appears to independently explain about 4% of the variance. It is not surprising that chronological age is such a strong predictor of processing speed and perceptual organization as Salthouse has long argued that age-related cognitive decline is mediated primarily by changes in cognitive processing speed whether measured directly or not (Salthouse, 1985, 1996, 2010a). For the regression analysis of the WAIS Verbal Comprehension factor score, the regression equation was significant, *F*(6, 91) = 2.36, *p* < .05, but explained only 7.7% of the variance (adjusted *r^2^*). The standardized beta coefficients are shown, together with the *t* test of each beta coefficient and various correlations, in the middle portion of Table 3. The lone significant predictor was T1 temporal-order performance, which accounted for about 10% of the variance (unadjusted). Finally, for the regression analysis of the T2 WAIS Working Memory factor scores, the regression equation was again significant, *F*(6, 91) = 2.54, *p* < .05, with the T1 independent variables explaining 8.7% of the variance (adjusted *r^2^*). The standardized beta coefficients are shown, together with the *t* test for those coefficients and various correlations, in the bottom portion of Table 3. Two significant predictors were identified, high-frequency hearing loss in both ears (T1 PC hearing loss at and above 2000 Hz bilaterally) and temporal-order performance (T1 PC temporal-order identification), with slightly greater contributions from the hearing loss as reflected by the partial and part (semipartial) correlations in Table 3. In summary, for all three T2 WAIS-III principal components, auditory temporal-order processing at T1, 9 years earlier, emerged as a significant predictor. Again, the amount of variance in T2 cognitive performance explained by auditory temporal processing is small, generally less than 10%. As noted previously, however, these significant, but small, contributions are consistent with the prior literature on the associations between sensory and cognitive function in older adults. It is noteworthy that baseline T1 hearing loss only emerged as a significant factor in the analysis of T2 WAIS-III Working Memory because most prior longitudinal studies examining the association between auditory and cognitive function made use of clinical audiograms as their only auditory measure.

**Table 3.** Results of multiple linear regression analyses with baseline (T1) auditory principal component (PC) factor scores and *z*-transformed age as the independent variables and the dependent measures of follow-up (T2) principal component (PC) factor scores for: (top) Wechsler Adult Intelligence Scale–Third Edition (WAIS-III) Processing Speed/Perceptual Organization (W3 PC PSPO), (middle) WAIS-III Verbal Comprehension (W3 PC VC), and (bottom) WAIS-III Working Memory (W3 PC WM).

Dependent variable: T2PC W3 PSPO	Standardized	*t*	Sig.	Correlations		
	Beta			Zero-order	Partial	Part
(Constant)		0.000	1.000			
T1Zage	−.605	−5.861	**.000**	−.628	−.523	−.465
T1PC HFHLbil	.056	0.597	.552	−.245	.062	.047
T1PC LMFHLrt	−.038	−0.447	.656	−.226	−.047	−.035
T1PC LMFHLlt	.021	0.265	.792	−.036	.028	.021
T1PC TempOrd	−.168	−2.028	**.046**	−.313	−.208	−.161
T1PC GapDet	.023	0.285	.776	.033	.030	.023
**Dependent Variable: T2PC W3 VC**	**Standardized**	***t***	**Sig.**	**Correlations**		
	**Beta**			**Zero-order**	**Partial**	**Part**
(Constant)		0.000	1.000			
T1Zage	.084	0.664	.508	−.003	.069	.065
T1PC HFHLbil	.063	0.542	.589	.105	.057	.053
T1PC LMFHLrt	−.127	−1.203	.232	−.100	−.125	−.117
T1PC LMFHLlt	−.040	−0.402	.689	−.031	−.042	−.039
T1PC TempOrd	−.325	−3.179	**.002**	−.305	−.316	−.310
T1PC GapDet	−.123	−1.261	.210	−.125	−.131	−.123
**Dependent Variable: T2PC W3 WM**	**Standardized**	***t***	**Sig.**	**Correlations**		
	**Beta**			**Zero-order**	**Partial**	**Part**
(Constant)		0.000	1.000			
T1Zage	.098	0.775	.441	−.128	.081	.075
T1PC HFHLbil	−.330	−2.850	**.005**	−.281	−.286	−.277
T1PC LMFHLrt	−.006	−0.061	.952	.024	−.006	−.006
T1PC LMFHLlt	−.001	−0.009	.993	.008	−.001	−.001
T1PC TempOrd	−.254	−2.496	**.014**	−.230	−.253	−.242
T1PC GapDet	−.069	−0.710	.479	−.071	−.074	−.069

*Note.* Significant Beta values have the significance level highlighted in bold font. Sig. = significance; T1Zage = the *z*-transformed age at baseline (T1); HFHLbil = hearing loss at and above 2000 Hz bilaterally; LMFHLrt = hearing loss below 2000 Hz in the right ear; LMFHLlt = hearing loss below 2000 Hz in the left ear; TempOrd = temporal-order identification; GapDet = gap detection.

## General Summary and Conclusions

The group data exhibited significant declines in mean hearing thresholds, whether measured clinically or psychophysically in the laboratory, mean gap-detection thresholds at 3500 Hz, and mean processing-related cognitive function as measured by the WAIS-III over the 9-year baseline (T1) to follow-up (T2) interval in this group of 98 older adults. Of these longitudinal declines, only the decline in gap-detection threshold had not been reported previously and represents a new finding. The other measures of temporal processing in this study, all making use of brief vowels in several variations of a temporal-order identification task, did not show significant declines in *average* performance over the 9-year period. In fact, the monaural two-item task (Mono2) showed a slight but significant improvement in mean performance.

When the individual data were examined in terms of slopes or rates of change over the 9-year period, several significant but relatively weak negative partial correlations (−.21 ≤ *r_p_* ≤ −.32) emerged between auditory measures of temporal-order identification and cognitive function. As noted, the analysis of the original cross-sectional data of Humes, Busey, et al. (2013) by Danielsson et al. (2019) reported the links between auditory function and cognition were strongest for auditory temporal-order processing and this is confirmed here for the longitudinal data. Given the nature of the measures involved, a negative correlation implies an association between the rate of decline in temporal processing and the rate of decline in cognitive function over the 9-year period. That is, the trajectory of declines over the 9-year period for these temporal-order and cognitive measures was similar, even when controlling for age, and points to possible shared underlying causes. Of the eight negative correlations observed among the auditory and cognitive slopes, six involved a dichotic measure of temporal-order processing. Only one negative correlation between monaural temporal-order identification and cognitive function emerged. The monaural two-item temporal-order identification and its dichotic counterpart are very similar tasks, but the dichotic version taps both additional attentional resources, dividing attention between ears within a trial, and binaural processing. It is likely that one or both additional processing mechanisms underlies the stronger association between, and the concomitant decline of, dichotic temporal-order performance and cognitive function. It is also noteworthy that rates of decline for the other psychophysical auditory measures, hearing loss and gap-detection, showed basically no association with the rate of cognitive decline except for one significant negative correlation (*r* = −.25) between the threshold at 4000 Hz and a measure of working memory (Letter–Number Sequence test).

Multiple regression analyses examined individual differences in the influence of baseline (T1) auditory performance on subsequent 9-year follow-up (T2) cognitive performance. At T2, both the full WAIS-III and several brief clinical tests were administered to assess cognition. When age, hearing loss, gap detection, and temporal-order identification at T1 were included as independent variables in the multiple regression analyses, the most consistent T1 predictor of T2 cognitive performance was temporal-order identification. This factor emerged as a significant predictor in all three T2 WAIS-III analyses and in one of the three analyses of brief cognitive tests (MMSE; Humes, 2020). In contrast, hearing loss emerged as a significant T1 predictor of T2 cognition only once (WAIS-III Working Memory).

Across all correlational and multiple regression analyses of T1–T2 slopes or T1 prediction of T2 cognition, typically, a total of 10%–12% of the variance was accounted for by one or more auditory measures. Although statistically significant in each analysis, this is a relatively small percentage of the variance in T1–T2 cognitive change or T2 cognitive function. Nonetheless, these percentages are consistent with recent systematic reviews of the association between auditory function and cognitive decline (e.g., Livingston et al., 2017; Loughrey et al., 2018). The biggest difference here, however, is that the only measure of auditory function included in prior studies and in these systematic reviews was the audiogram. In this study, hearing loss only emerged as a significant explanatory factor for measures of working memory. Here, the auditory measure that typically accounted for the most variance in cognitive function, whether T1–T2 rates of change or T1 predictors of T2 performance, was temporal-order identification, confirming trends in analyses of the cross-sectional data (Danielsson et al., 2019; Humes, Busey, et al., 2013). The present linear-regression analyses (see Table 3) with six predictors (age and five auditory factors) could detect significant regression solutions with an *r* ^2^ of .13 with 80% power, (*p* = .05) and, as noted above, most regression analyses were significant. However, the sample size may have been too small to detect significant partial effects of hearing loss on cognitive function, which, based on the present analyses, were considerably smaller than the effects of temporal-order processing.

Good agreement was also observed between the average data over the adult life span from the original cross-sectional study (Humes et al., 2009, 2010; Humes, Busey, et al., 2013) and the longitudinal data presented here both for auditory and cognitive measures (see Figures 5 and 6). The correlations observed between sensory and cognitive function in the cross-sectional study of Humes, Busey, et al. (2013), however, were stronger than observed longitudinally here. Correlations between measures of auditory processing and cognitive function, *all from the T1 baseline*, were computed for the 98 participants who returned for this study. As in the previously reported associations among rates of decline (see Table 2) and between T1 auditory function and T2 cognitive function (see Table 3), the strongest associations were between the T1 temporal-order principal component and T1 cognitive function, with correlations of −.27, −.38, and −.30 for T1 WAIS-III Verbal Comprehension, PSPO, and Working Memory principal components, respectively. In addition, two significant negative correlations, −.24 and −.28, were observed between two of the T1 hearing-loss principal components and the T1 WAIS-III PSPO principal component. When regression analyses parallel to those summarized in Table 3 were conducted, this time with the *baseline T1* WAIS-III principal components as the dependent variables, the results were nearly identical to those shown in Table 3 both in terms of variance explained and the relative roles of each of the predictor variables. It is likely that the larger correlations observed between auditory and cognitive function in the earlier cross-sectional study were due to the much broader age range in that study, 18–87 years, than in this study (e.g., Hofer et al., 2006).

Finally, it is important to note that when links between auditory function and cognitive function were observed, whether between static T1 and T2 measures or for measures of rates of change from T1 to T2, the strength of the associations that emerged varied both with the auditory measures and the cognitive measures involved. This is consistent with prior work (e.g., Rönnberg et al., 2011 , 2014) as well as recent structural-equation modeling of cross-sectional (Danielsson et al., 2019) and longitudinal (Pronk et al., 2019) data. Although hearing researchers are well aware that there are multiple aspects to “auditory function,” it is not often appreciated that this is true for “cognitive function” as well, even *within* the so-called fluid or process-based cognitive measures and the crystallized or product-based cognitive measures. This was apparent in this study when the 13 scales of the WAIS-III were reduced to three principal components and that the strength of associations with age and auditory measures varied for each of these three cognitive components.

## References

[bib1] American National Standards Institute. (2003). Maximum permissible ambient noise levels for audiometric test rooms (ANSI S3.1-1999; R2003).

[bib2] American National Standards Institute. (2004). Specification for audiometers (ANSI S3.6-2004)).

[bib3] Babkoff, H. , & Fostick, L. (2017). Age-related changes in auditory processing and speech perception: Cross-sectional and longitudinal analyses. European Journal of Ageing, 14, 269–281. https://doi.org/10.1007/s10433-017-0410-y 28936137 10.1007/s10433-017-0410-y PMC5587455

[bib4] Bronkhorst, A. W. (2000). The cocktail party phenomenon: A review of speech intelligibility in multiple-talker conditions. Acta Acustica, 86, 117–128.

[bib5] Bronkhorst, A. W. (2015). The cocktail-party phenomenon revisited: Early processing and selection of multi-talker speech. Attention, Perception, & Psychophysics, 77, 1465–1487. https://doi.org/10.3758/s13414-015-0882-9 10.3758/s13414-015-0882-9 PMC4469089 25828463

[bib6] Cherry, E. C. (1953). Some experiments on the recognition of speech, with one and with two ears. The Journal of the Acoustical Society of America, 25(5), 975–979. https://doi.org/10.1121/1.1907229

[bib7] Danielsson, H. , Humes, L. E. , & Ronnberg, J. (2019). Different associations between audition and cognition depending on type of auditory function and type of cognition. Ear and Hearing, 40(5), 1210–1219. https://doi.org/10.1097/AUD.0000000000000700 30807540 10.1097/AUD.0000000000000700 PMC6706331

[bib8] Evans, A. S. (1978). Causation and disease: A chronological journey. American Journal of Epidemiology, 108(4), 248–255. https://doi.org/10.1093/oxfordjournals.aje.a112617 10.1093/oxfordjournals.aje.a112617 727194

[bib9] Fogerty, D. , Humes, L. E. , & Kewley-Port, D. (2010). Auditory temporal-order processing of vowel sequences by young and elderly listeners. The Journal of the Acoustical Society of America, 127(4), 2509–2520. https://doi.org/10.1121/1.3316291 20370033 10.1121/1.3316291 PMC2865703

[bib10] Folstein, M. F. , Folstein, S. E. , & McHugh, P. R. (1975). Mini-Mental State: A practical method for grading the cognitive state of patients for the clinician. Journal of Psychiatric Research, 12(3), 189–198. https://doi.org/10.1016/0022-3956(75)90026-6 1202204 10.1016/0022-3956(75)90026-6

[bib11] Fraisse, P. (1984). Perception and estimation of time. Annual Reviews in Psychology, 35, 1–36. https://doi.org/10.1146/annurev.ps.35.020184.000245 10.1146/annurev.ps.35.020184.000245 6367623

[bib12] Frank, T. , & Richards, W. D. (1991). Hearing aid coupler output level variability and coupler correction levels for insert earphones. Ear and Hearing, 12, 221–227. https://doi.org/10.1097/00003446-199106000-00010 1916048 10.1097/00003446-199106000-00010

[bib13] Gatehouse, S. , & Davis, A. (1992). Clinical pure-tone versus three-interval forced-choice thresholds: Effects of hearing level and age. International Journal of Audiology, 31(1), 31–44. https://doi.org/10.3109/00206099209072900 10.3109/00206099209072900 1554331

[bib14] Gates, G. A. , & Cooper, J. C. (1991). Incidence of hearing decline in the elderly. Acta Otolaryngologica, 111(2), 240–248. https://doi.org/10.3109/00016489109137382 10.3109/00016489109137382 2068909

[bib15] Gates, G. A. , Anderson, M. , Feeney, M. P. , McCurrey, S. , & Larson, E. B. (2008). Central auditory dysfunction in older people with memory impairment or Alzheimer's dementia. Archives of Otolaryngology—Head & Neck Surgery, 134(7), 771–777. https://doi.org/10.1001/archotol.134.7.771 18645130 10.1001/archotol.134.7.771 PMC2871110

[bib16] Gates, G. A. , Anderson, M. L. , McCurry, S. M. , Feeney, M. P. , & Larson, E. B. (2011). Central auditory dysfunction as a harbinger of Alzheimer's dementia. Archives of Otolaryngology—Head & Neck Surgery, 137(7), 390–395. https://doi.org/10.1001/archoto.2011.28 21502479 10.1001/archoto.2011.28 PMC3170925

[bib17] Gorsuch, R. L. (1983). Factor analysis (2nd ed.). Erlbaum.

[bib18] Hanna, T. E. , & Robinson, D. E. (1985). Phase effects for a sine wave masked by reproducible noise. The Journal of the Acoustical Society of America, 77(3), 1129–1140. https://doi.org/10.1121/1.392177 10.1121/1.392889 4056215

[bib19] Harris, K. C. , Eckert, M. A. , Ahlstrom, J. B. , & Dubno, J. R. (2010). Age-related differences in gap detection: Effects of task difficulty and cognitive ability. Hearing Research, 264(1–2), 21–29. https://doi.org/10.1016/j.heares.2009.09.017 19800958 10.1016/j.heares.2009.09.017 PMC2868108

[bib20] Hofer, S. M. , Flaherty, B. P. , & Hoffman, L. (2006). Cross-sectional analysis of time-dependent data: Mean-induced association in age-heterogeneous samples and an alternative method based on sequential narrow age-cohort samples. Multivariate Behavioral Research, 41, 165–187. https://doi.org/10.1207/s15327906mbr4102_4 26782909 10.1207/s15327906mbr4102_4

[bib21] Humes, L. E. (2007). The contributions of audibility and cognitive factors to the benefit provided by amplified speech to older adults. Journal of the American Academy of Audiology, 18(7), 609–623. https://doi.org/10.3766/jaaa.18.7.6 10.3766/jaaa.18.7.6 18236646

[bib60] Humes, L. E. (2020). Associations between measures of auditory function and brief assessments of cognitive function. American Journal of Audiology, 29(4), 825–837. https://doi.org/10.1044/2020_AJA-20-00077 32976027 10.1044/2020_AJA-20-00077 PMC8608158

[bib22] Humes, L. E. , Busey, T. A. , Craig, J. C. , & Kewley-Port, D. (2009). The effects of age on sensory thresholds and temporal gap detection in hearing, vision and touch. Attention, Perception, & Psychophysics, 71, 860–871. https://doi.org/10.3758/APP.71.4.860 10.3758/APP.71.4.860 PMC2826883 19429964

[bib23] Humes, L. E. , Busey, T. A. , Craig, J. , & Kewley-Port, D. (2013). Are age-related changes in cognitive function driven by age-related changes in sensory processing? Attention, Perception, & Psychophysics, 75, 508–524. https://doi.org/10.3758/s13414-012-0406-9 10.3758/s13414-012-0406-9 PMC3617348 23254452

[bib24] Humes, L. E. , & Dubno, J. R. (2010). Factors affecting speech understanding in older adults. In S. Gordon-Salant , R. D. Frisina , A. N. Popper , & R. R. Fay (Eds.), The aging auditory system: Perceptual characterization and neural bases of presbycusis. Springer.

[bib25] Humes, L. E. , Dubno, J. R. , Gordon-Salant, S. , Lister, J. J. , Cacace, T. , Cruickshanks, K. , Gates, G. , Wilson, R. , & Wingfield, A. (2012). Central presbycusis: A review and evaluation of the evidence. Journal of the American Academy of Audiology, 23(8), 635–666. https://doi.org/10.3766/jaaa.23.8.5 22967738 10.3766/jaaa.23.8.5 PMC5898229

[bib26] Humes, L. E. , Kewley-Port, D. , Fogerty, D. , & Kinney, D. (2010). Measures of hearing threshold and temporal processing across the adult lifespan. Hearing Research, 264(1–2), 30–40. https://doi.org/10.1016/j.heares.2009.09.010 19786083 10.1016/j.heares.2009.09.010 PMC3182849

[bib27] Humes, L. E. , Kidd, G. R. , & Lentz, J. J. (2013). Auditory and cognitive factors underlying individual differences in aided speech-understanding among older adults. Frontiers in Systems Neuroscience, 7(55), 1–16. https://doi.org/10.3389/fnsys.2013.00055 24098273 10.3389/fnsys.2013.00055 PMC3787592

[bib28] Humes, L. E. , & Young, L. A. (2016). Sensory-cognitive interactions in older adults. Ear and Hearing, 37, 52S–61S. https://doi.org/10.1097/AUD.0000000000000303 27355770 10.1097/AUD.0000000000000303 PMC4930008

[bib29] James, W. (1890). The principles of psychology (Vol. 1). Holt. https://doi.org/10.1037/10538-000

[bib30] Kawahara, H. , Masuda-Kastuse, I. , & Cheveigne, A. (1999). Restructuring speech representations using a pitch-adaptive time-frequency smoothing and an instantaneous-frequency-based F0 extraction: Possible role of a repetitive structure in sounds. Speech Communication, 27(3–4), 187–207. https://doi.org/10.1016/S0167-6393(98)00085-5

[bib31] Lee, F.-S. , Matthews, L. J. , Dubno, J. R. , & Mills, J. H. (2005). Longitudinal study of pure-tone thresholds in older persons. Ear and Hearing, 26(1), 1–11. https://doi.org/10.1097/00003446-200502000-00001 15692300 10.1097/00003446-200502000-00001

[bib32] Levitt, H. (1971). Transformed up-down method in psychoacoustics. The Journal of the Acoustical Society of America, 49(2B), 467–477. https://doi.org/10.1121/1.1912375 5541744

[bib33] Lin, F. R. , Ferrucci, L. , Metter, E. J. , Yang, A. , Zonderman, A. B. , & Resnick, S. M. (2011). Hearing loss and cognition in the Baltimore longitudinal study of aging. Neuropsychology, 25(6), 763–770. https://doi.org/10.1037/a0024238 21728425 10.1037/a0024238 PMC3193888

[bib34] Lin, F. R. , Metter, E. J. , O'Brien, R. J. , Resnick, S. M. , Zonderman, A. B. , & Ferrucci, L. (2011). Hearing loss and incident dementia. Archives of Neurology, 68(2), 214–220. https://doi.org/10.1001/archneurol.2010.362 21320988 10.1001/archneurol.2010.362 PMC3277836

[bib35] Lin, F. R. , Yaffe, K. , Xia, J. , Xue, Q.-L. , Harris, T. B. , Purchase-Helzner, E. , Satterfield, S. , Ayonayon, H. N. , Ferrucci, L. , Simonsick, E. M. , & for the Health ABC Study Group. (2013). Hearing loss and cognitive decline in older adults. JAMA Internal Medicine, 173(4), 293–299. https://doi.org/10.1001/jamainternmed.2013.1868 23337978 10.1001/jamainternmed.2013.1868 PMC3869227

[bib36] Lindenberger, U. , & Baltes, P. B. (1994). Sensory functioning and intelligence in old age: A strong connection. Psychology and Aging, 9(3), 339–355. https://doi.org/10.1037/0882-7974.9.3.339 7999320 10.1037//0882-7974.9.3.339

[bib37] Livingston, G. , Sommerlad, A. , Orgeta, V. , Costafreda, S. G. , Huntley, J. , Ames, D. , Ballard, C. , Banerjee, S. , Burns, A. , Cohen-Mansfield, J. , Cooper, C. , Fox, N. , Gitlin, L. N. , Howard, R. , Kales, H. C. , Larson, E. B. , Ritchie, K. , Rockwood, K. , Sampson, E. L. , … Mukadam, N. (2017). Dementia prevention, intervention, and care. Lancet, 390(10113), 2673–2734. https://doi.org/10.1016/S0140-6736(17)31363-6 28735855 10.1016/S0140-6736(17)31363-6

[bib38] Loughrey, D. G. , Kelly, M. E. , Kelley, G. A. , Brennan, S. , & Lawlor, B. A. (2018). Association of age-related hearing loss with cognitive function, cognitive impairment, and dementia: A systematic review and meta-analysis. JAMA Otolaryngology—Head & Neck Surgery, 144(2), 115–126. https://doi.org/10.1001/jamaoto.2017.2513 29222544 10.1001/jamaoto.2017.2513 PMC5824986

[bib39] Marshall, L. (1991). Decision criteria for pure-tone detection used by two age groups of normal-hearing and hearing-impaired listeners. Journal of Gerontology, 46(2), 67–70. https://doi.org/10.1093/geronj/46.2.P67 10.1093/geronj/46.2.p67 1997578

[bib40] Marshall, L. , & Jesteadt, W. (1986). Comparison of pure-tone audibility thresholds obtained with audiological and two-interval forced-choice procedures. Journal of Speech and Hearing Research, 29(1), 82–91. https://doi.org/10.1044/jshr.2901.82 3702383 10.1044/jshr.2901.82

[bib41] Pearson, J. D. , Morrell, C. H. , Gordon-Salant, S. , Brant, L. J. , Metter, E. J. , Klein, L. L. , & Fozard, J. L. (1995). Gender differences in a longitudinal study of age-associated hearing loss. The Journal of the Acoustical Society of America, 97(2), 1196–1205. https://doi.org/10.1121/1.412231 7876442 10.1121/1.412231

[bib42] Potash, M. , & Jones, B. (1977). Aging and decision criteria for the detection of tones in noise. Journal of Gerontology, 32(4), 436–440. https://doi.org/10.1093/geronj/32.4.436 864208 10.1093/geronj/32.4.436

[bib43] Pronk, M. , Lissenberg-Witte, B. I. , van der Aa, H. P. A. , Comjis, H. C. , Smits, C. , Lemke, U. , Zekveld, A. A. , & Kramer, S. E. (2019). Longitudinal relationships between decline in speech-in-noise recognition ability and cognitive functioning: The longitudinal aging study in Amsterdam. Journal of Speech, Language, and Hearing Research, 62(4S), 1167–1187. https://doi.org/10.1044/2018_JSLHR-H-ASCC7-18-0120 10.1044/2018_JSLHR-H-ASCC7-18-0120 31026198

[bib44] Rees, J. N. , & Botwinick, J. (1980). Detection and decision factors in auditory behavior of the elderly. Journal of Gerontology, 26(2), 133–136. https://doi.org/10.1093/geronj/26.2.133 10.1093/geronj/26.2.133 5554313

[bib45] Rönnberg, J. , Danielsson, H. , Rudner, M. , Arlinger, S. , Sternäng, O. , Wahlin, Å. , & Nilsson, L.-G. (2011). Hearing loss is negatively related to episodic and semantic long-term memory but not to short-term memory. Journal of Speech, Language, and Hearing Research, 54, 705–726. https://doi.org/10.1044/1092-4388(2010/09-0088) 10.1044/1092-4388(2010/09-0088) 20884779

[bib46] Rönnberg, J. , Hygge, S. , Keidser, G. , & Rudner, M. (2014). The effect of functional hearing loss and age on long- and short-term visuospatial memory: Evidence from the UK Biobank resource. Frontiers in Aging Neuroscience, 6, 326. https://doi.org/10.3389/fnagi.2014.00326 25538617 10.3389/fnagi.2014.00326 PMC4260513

[bib47] Ronen, M. , Lifshitz-Ben-Basat, A. , Taitelbaum-Swead, R. , & Fostick, L. (2018). Auditory temporal processing, reading, and phonological awareness among aging adults. Acta Psychologica, 190, 1–10. https://doi.org/10.1016/j.actpsy.2018.06.010 29986206 10.1016/j.actpsy.2018.06.010

[bib48] Salthouse, T. A. (1985). A theory of cognitive aging. North-Holland.

[bib49] Salthouse, T. A. (1996). The processing-speed theory of cognitive aging. Psychological Review, 103(3), 403–428. https://doi.org/10.1037/0033-295X.103.3.403 8759042 10.1037/0033-295x.103.3.403

[bib50] Salthouse, T. A. (2010a). Major issues in cognitive aging. Oxford University Press.

[bib51] Salthouse, T. A. (2010b). Selective review of cognitive aging. Journal of the International Neuropsychological Society, 16(5), 754–760. https://doi.org/10.1017/S1355617710000706 20673381 10.1017/S1355617710000706 PMC3637655

[bib52] Salthouse, T. A. (2011). Effects of age on time-dependent cognitive change. Psychological Science, 22(5), 682–688. https://doi.org/10.1177/0956797611404900 21467547 10.1177/0956797611404900 PMC3631712

[bib53] Schaie, K. W. (1983). Longitudinal studies of adult psychological development. Guilford Press.

[bib54] Schaie, K. W. (2005). Developmental influences on adult intelligence: The Seattle Longitudinal Study. Oxford University Press.

[bib55] Schneider, B. A. , & Pichora-Fuller, M. K. (2000). Implications of perceptual processing for cognitive aging research. In F. I. M. Craik & T. A. Salthouse (Eds.), The handbook of aging and cognition (2nd ed.). Erlbaum.

[bib56] Wayne, R. V. , & Johnsrude, I. S. (2015). A review of causal mechanisms underlying the link between age-related hearing loss and cognitive decline. Ageing Research Review, 23(Pt. B), 154–166. https://doi.org/10.1016/j.arr.2015.06.002 10.1016/j.arr.2015.06.002 26123097

[bib57] Wechsler, D. (1997). Wechsler Adult Intelligence Scale–Third Edition (WAIS-III). The Psychological Corporation. https://doi.org/10.1037/t49755-000

[bib58] Wechsler, D. (2008). Wechsler Adult Intelligence Scale–Fourth Edition (WAIS-IV). Pearson. https://doi.org/10.1037/t15169-000

[bib59] Wiley, T. L. , Chappell, R. , Carmichael, L. , Nondahl, D. M. , & Cruickshanks, K. J. (2008). Changes in hearing thresholds over 10 years in older adults. Journal of the American Academy of Audiology, 19(4), 281–292. https://doi.org/10.3766/jaaa.19.4.2 18795468 10.3766/jaaa.19.4.2 PMC2802451

